# Responsible Design Thinking for Sustainable Development: Critical Literature Review, New Conceptual Framework, and Research Agenda

**DOI:** 10.1007/s10551-023-05600-z

**Published:** 2024-02-05

**Authors:** Brian Baldassarre, Giulia Calabretta, Ingo Oswald Karpen, Nancy Bocken, Erik Jan Hultink

**Affiliations:** 1 Faculty of Industrial Design Engineering, Delft University of Technology, Delft, The Netherlands; 2 School of Business and Economics, Maastricht Sustainability Institute, Maastricht University, Maastricht, The Netherlands; 3 CTF Service Research Center, Karlstad University, Karlstad, Sweden; 4 Adelaide Business School, University of Adelaide, Adelaide, Australia

**Keywords:** Design, Responsible innovation, Responsible business, Circular economy, Sustainable innovation, Sustainability

## Abstract

In the 1960s, influential thinkers defined design as a rational problem-solving approach to deal with the challenges of sustainable human development. In 2009, a design consultant and a business academic selected some of these ideas and successfully branded them with the term “design thinking.” As a result, design thinking has developed into a stream of innovation management research discussing how to innovate faster and better in competitive markets. This article aims to foster a reconsideration of the purposes of design thinking moving forward, in view of the sustainable development challenges intertwined with accelerating innovation in a perpetual economic growth paradigm. To this end, we use a problematization method to challenge innovation management research on design thinking. As part of this method, we first systematically collect and critically analyze the articles in this research stream. We uncover a prominent focus on economic impact, while social and environmental impacts remain largely neglected. To overcome this critical limitation, we integrate design thinking with responsible innovation theorizing. We develop a framework for responsible design thinking, explaining how to apply this approach beyond a private interest and competitive advantage logic, to address sustainable development challenges, such as climate change, resource depletion, poverty, and injustice. The framework contributes to strengthening the practical relevance of design thinking and its theoretical foundations. To catalyze this effort, we propose an agenda for future research.

**Supplementary Information:**

The online version contains supplementary material available at 10.1007/s10551-023-05600-z.

## Introduction

World War II was followed by an economic boom. Production and consumption intensified quickly. Scientists started to point out that such rapid growth may not be sustained in the long run, without incurring into a global environmental and societal crisis (Carson, 1962; Fuller, 1969; Hardin, 1968). In 1969, humans went to the moon and, for the first time, a picture of the Earth was taken from far away enough, to clearly see that our planet is relatively small and isolated in the universe, leaving nowhere else to go in case of a systemic collapse. Buckminster Fuller, an architect and influential thinker, started to talk about a “Comprehensive Anticipatory Design Science” that could be adopted to define an “Operating Manual for Spaceship Earth” (Fuller, 1957, 1969). This was urgently needed to “design for the real world” (Papanek, 1971), dealing with the emerging crisis by optimizing the use of resources, ensuring their fair distribution, managing waste, and pollution (Carson, 1962; Fuller, 1969; Hardin, 1968).

Against this background, Herbert Simon, who was later awarded the Nobel Prize in economics for his ideas on rationality and decision-making, investigated design science as a process to create solutions for complex problems, including those systemic issues affecting society and the environment (Simon, 1968). Rittel and Webber (1973) further elaborated on the nature of these problems, arguing that they are “wicked” because they cannot be solved in the same context where they originated. Throughout the 1980s and 1990s, several academics elaborated on the role that designers may play in addressing these problems. This role can be boiled down to “reflecting in action”: experimentally introducing and improving new visual artifacts, products, services, buildings and urban environments to enhance the lives of people and society (Archer, 1979; Buchanan, 1992; Cross, 1982, 2007; Schön, 1983, 1992). More recently, “the business community has adapted design thinking from [these] engineering and architecture [views]” (p. 91), using it as an “umbrella term” (p. 92) to describe a problem-solving approach for innovating organizations (Hamington, 2019). Brown (2008) and Martin (2009) played a key role in this adaptation process (Brown & Martin, 2015). Neglecting some key environmental and social considerations in the original ideas, and putting emphasis on innovation with a business mindset, they created the label “design thinking” (Dunne & Martin, 2006). This discussion around “design thinking” progressively consolidated into a stream of innovation management literature (Verganti, 2017; Verganti et al., 2021).

Innovation management has developed over the last half century into a mature field of research with many dedicated high-quality journals and conferences (Linton & Thongpapanl, 2004). This field investigates how organizations depend on the successful development and introduction of new products and services to survive in the long term. Recurrent and enduring challenges of innovation management are, for instance, balancing innovation portfolios, dealing with disruption, integrating organizational, technological, and commercial priorities, building advantage through intangible assets and activities, and encouraging creativity (Dodgson et al., 2013). Multiple studies have investigated trends, drivers, and best practices in innovation management (e.g., Barczak et al., 2009) and several academics offered recommendations for the application of research methodologies to investigate innovation management in a valid and reliable manner (e.g., Goffin et al., 2019). Recent specializations within the innovation management field are, for example, open innovation, data-enabled innovation, and design thinking.

Within this innovation management field, design thinking has indeed crystallized as a stream of research discussed by a community of scholars across several journals and academic conferences (Verganti et al., 2021). Design thinking is defined as an experimental, user-centered, and collaborative approach to solve wicked problems (Brown, 2009; Martin, 2009). This research stream emphasizes that “doing design thinking” relies on a set of practices that organizations can perform to accelerate the innovation process, achieve competitive advantage, and improve the economic performance of innovations (Elsbach & Stigliani, 2018; Micheli et al., 2019). While economic impact is indeed an important aspect, it is becoming increasingly clear that innovation management must consider environmental and social impacts as well (Elkington, 1998; Voegtlin & Scherer, 2017). For instance, accelerating innovation in the smartphone industry is linked to depletion of critical materials, deforestation, violation of human rights in developing countries, and rising greenhouse emissions (Akemu et al., 2016; Biedenkopf et al., 2019). On these grounds, Hamington (2019) recently stated that “design thinking needs a moral corollary” (p. 92) and proposed to integrate this stream of innovation management literature on design thinking with the business ethics literature. In a similar vein, Greenwood and Freeman (2018) pointed out that “many business innovation frameworks, from sophisticated theory published in top journals to ideas used in consulting and problem solving, could be improved and deepened by surfacing otherwise implicit ethical analysis” (p. 3).

Aiming to address these calls to action from a design thinking perspective, we turn to the business ethics literature on responsible innovation (de los Reyes & Scholz, 2022; Steen et al., 2021; Voegtlin & Scherer, 2017). The origins of this approach relate to the ideas of Jürgen Habermas, who discussed deliberative democracy and procedural justice (de Hoop et al., 2016). More recently, these ideas gained momentum in European policy-making, as addressing the environmental and social challenges of sustainable development became a cross-cutting issue under the European Framework Program for Research and Innovation Horizon 2020 (European Commission, 2013, 2018; de Hoop et al., 2016; Mazzucato, 2018; Von Schomberg, 2013). These challenges, such as climate change, resource depletion, poverty, and injustice, are essentially wicked problems, since they are interlinked and cannot be solved without systemic changes (Ferraro et al., 2015; George et al., 2015, 2016; Reinecke & Ansari, 2016). Blok and Lemmens (2015) provide an example related to the energy production problem: producing biofuels may reduce greenhouse emissions contributing to climate change, but at the same time, it requires the use of land in developing countries, affecting local food supply chains, and leading to an international conflict of interest, in which not all stakeholders have the same decision-making power.

This discussion permeated from the policy domain into a stream of business ethics literature, due to the increasing pressure on business organizations to mitigate their negative impacts in a globalized world (Scherer & Palazzo, 2011). Business ethics is a research field that has grown and developed into a large body of knowledge, discussed in dedicated journals such as the *Journal of Business Ethics* (Calabretta et al., 2011), focusing on related research topics such as ethical judgments (Sparks & Pan, 2010), international business ethics, and environmental ethics (Fassin, 2000; Werhane & Freeman, 1999). Responsible innovation has become an important stream within the field of business ethics (Pandza & Ellwood, 2013; Voegtlin & Scherer, 2017). The responsible innovation literature elaborates on the features and conceptual dimensions of the approach from an organizational perspective (Burget et al., 2017; de los Reyes & Scholz, 2022; Gutierrez et al., 2022; Lubberink et al., 2017). In this context, responsible innovation is defined as an experimental and collaborative approach of adaptive learning to solve wicked problems, with an ethical focus on economic, social, and environmental impacts (Voegtlin & Scherer, 2017; Von Schomberg, 2013).

Innovation management literature on design thinking and business ethics literature on responsible innovation present similar yet divergent approaches. Both approaches are based on an experimental process to address wicked problems from an organizational perspective. However, innovation management scholars discuss design thinking with a user-centered yet narrow focus on competitive advantage and economic impact, while business ethics scholars discuss responsible innovation in view of holistic and ethical considerations geared toward achieving social, environmental, and economic impacts simultaneously. Considering these structural similarities and divergences in scope, the objective of this article is to integrate the two approaches, advancing innovation management research on design thinking with a view on business ethics (Hamington, 2019).

As a result, we propose a novel framework for what we call “responsible design thinking,” linking specific design thinking practices with conceptual dimensions of responsible innovation. We substantiate ethical thinking in innovation management by drawing on responsibility literature (e.g., Stilgoe et al., 2013; Voegtlin & Scherer, 2017), and reposition design thinking to bring back more of its original purpose of doing innovation for the benefit of society and the natural environment, rather than just for competitive advantage. This revived connection contributes to addressing ethical and sustainable limitations of innovation management research on design thinking. At a foundational level, this stream of literature has been criticized for lacking theoretical grounding and unfolding “in a self-referential vacuum” (Johansson-Sköldberg et al., 2013; Verganti et al., 2021). Recent work suggests addressing the issue by connecting to other innovation theories (Gemser & Barczak, 2020; Verganti et al., 2021). Our link to responsible innovation advances these calls, providing theoretical grounding for design thinking practices, and conceptually explaining why and how they may be applied to address sustainable development challenges. At a higher level, infusing responsible insights into the innovation management literature on design thinking serves to recalibrate its scope with a view on business ethics, as explicitly requested by recent articles (Greenwood & Freeman, 2018; Hamington, 2019; Steen et al., 2021). The scope is broadened beyond a myopic focus on economic impact, which largely ignores the social and environmental side effects of innovation (Staton et al., 2016). Work in this sense is slowly emerging, although it is still limited and scattered (e.g., Baldassarre, 2021; Baldassarre et al., 2019; Bason & Austin, 2019; Hamington, 2019; Liedtka et al., 2017). Acknowledging the importance of these efforts, we leverage our proposed framework by putting forward a research agenda on responsible design thinking.

To develop the framework and research agenda, we use a rigorous problematization method (Alvesson & Sandberg, 2011), which is introduced and explained in the next section.

## Problematization Method

Problematization is a structured method to look critically at existing theories or literature streams and accordingly generate insights to guide future research (Alvesson & Sandberg, 2011, 2013, 2020). Using the words of Alvesson and Sandberg, the idea is to enable “an ‘opening up’ rather than a ‘building exercise,’ catalyzing new conversations rather than just continuing old ones” (Alvesson & Sandberg, 2020, p. 1291). To this end, the method does not focus on “gap-spotting” or “filling a gap,” “in order to extend a literature” while leaving its underlying assumptions unchallenged, but rather on looking critically at these assumptions to construct new theories (Alvesson & Sandberg, 2011, p. 247–248). The method essentially entails selecting a literature stream, critically reviewing it to identify and challenge underlying assumptions, and ultimately formulating new assumptions, frameworks, and research questions to guide its future development (Alvesson & Sandberg, 2011, 2013, 2020).

The method has already been applied by scholars to open new conversations driven by ethical considerations. For example, Okimoto (2014) has used the method in the field of social justice studies to problematize extant literature on retributive justice, while Matthews et al. (2016) used it to uncover inherent tensions within sustainable supply chain management. The method has also been used at the intersection between innovation management and business ethics research (Danatzis et al., 2022; Greenwood & Freeman, 2018). Greenwood and Freeman (2018) argued for the use of problematization to question narrow business views and innovation theories based on biased Western canons: “To be ethical, we need also to take responsibility for our conceptual frameworks and their embedded assumptions” (p. 1). In this spirit, we problematize innovation management research on design thinking to foster more responsible investigations on the subject.

Problematization is based on six steps: (1) identifying the literature stream and its key texts; (2) uncovering the current assumption(s) in the literature stream; (3) evaluating the current assumption; (4) proposing an alternative assumption; (5) relating the alternative assumption to its audience; and (6) reflecting upon the alternative assumption to guide future research.

The remainder of this article is structured according to the method and its six main steps. The first three steps of problematization are covered in the next section “challenging the innovation management literature on design thinking.” We systematically collected and critically analyzed the entire body of innovation management literature on design thinking. As a result, we identified a core underlying assumption and evaluated it through a series of discussions with academics and innovation practitioners, to unravel where its problematic aspects lie. The fourth step of problematization is covered in the section “comparative analysis of design thinking and responsible innovation approaches.” We departed from the identification of the problematic aspects in the assumption to introduce the literature on responsible innovation, compare it with design thinking, and ultimately argue for an integration of both approaches. We proposed an alternative assumption to open a new research avenue responding to former requests for infusing business ethics into design thinking. The fifth step of problematization is covered in the section “framework for responsible design thinking.” We related the alternative assumption to the audiences of innovation management and business ethics scholars by articulating a conceptual framework through a process of conceptual integration of key elements from design thinking and responsible innovation. Finally, the sixth step of problematization is covered in the section “discussion and research agenda.” We performed brainstorming sessions and expert workshops to reflect upon the framework and alternative assumption and to generate relevant questions for future research on responsible design thinking.

## Challenging the Innovation Management Literature on Design Thinking (Problematization Steps 1, 2, 3)

This section covers the first three steps of the problematization method. We identify the literature stream and its key texts; we uncover the current assumption in the literature stream; and we evaluate the current assumption.

In line with the guidelines of Alvesson and Sandberg (2013, 2020), the first step of our study was to focus the scope of our analysis upon the stream of innovation management literature on design thinking and to identify the key texts. The origins of innovation management literature on design thinking are connected to increasing saturation, competition, and pressure to innovate in the markets of industrialized countries (Prahalad, 2012; Verganti, 2017). In 2008, British designer Tim Brown and the global consultancy firm IDEO were successful in interpreting the current situation. Loosely building upon former design science theories, they created the label “design thinking” and promoted it in business innovation practice as a more experimental and user-centered approach, with a strong focus on solving consumers’ problems faster and better (Brown, 2008; Hamington, 2019). Design thinking ideas quickly spread (Kolko, 2015; Martin, 2010). Many established organizations, such as IBM, Toyota, and 3 M, embraced it, while innovation consultancies incorporated them into their service portfolio (Liedtka et al., 2013). In parallel, behind well-marketed consultancy formulas, business academics started to focus on design thinking as well. In 2009, Roger Martin, an influential scholar, cherry picked certain elements from the design literature on problem-solving in the fields of engineering and architecture (e.g., Buchanan, 1992; Simon, 1995), re-elaborated them from a business perspective in alignment with Brown, and published a book that promoted the idea that “design thinking is the new competitive advantage” for firms (Martin, 2009). This idea gained momentum, leading to the consolidation of an innovation management literature stream on design thinking.

This process of consolidation is ongoing, and it has been driven by several authors (Verganti, 2008, 2009; Verganti et al., 2021). For example, Liedtka (2015) linked design thinking to superior innovation performance through cognitive bias reduction, while Micheli et al. (2018) discussed the competitive and industry conditions needed for elevating design to a strategic function. Elsbach and Stigliani (2018) explained how infusing design thinking into an organizational culture accelerates experiential learning, ultimately fostering a competitive advantage. Kumar and Holloway (2009) argued that design thinking can be used to improve partnerships and channel strategies, as well as portfolio management and the revenue model for an organization to grow. In a similar vein, Kolko (2015) explained that large firms may use design thinking to globalize their business, and Beverland et al. (2015) discussed the relevance of design thinking as a mechanism to sustain growth and brand equity. Gruber et al., (2015, p. 1) describe successful firms that have “exploited design to translate technological innovation into products that deliver compelling customer experiences and have come to dominate their respective industry sectors.” Zooming out, Verganti et al. (2021) recently described design thinking as a “paradigm” within the innovation management literature. These examples are relevant to point out that innovation management scholars extensively studied the economic impact of applying design thinking. However, it also became clear that economic impact cannot be pursued at the expense of society and the environment, which may collapse under the stress of unsustainable growth (Meadows et al., 1972; Rockström et al., 2009).

In recent years, a few scholars started a conversation about design thinking in relation to societal issues. For example, Liedtka et al. (2017) wrote a book on “design thinking for the greater good,” illustrating case studies of innovation in the areas of health care, agriculture, transportation, social services, and security, performed both by governments and by business organizations. Bason and Austin (2019) examined the application of design thinking in the public sector to make a positive social impact. To better understand if and how innovation management scholars investigated the impact of design thinking beyond an economic sense, also considering impacts on society and the environment, we systematically conducted a literature review with a critical lens.

**Fig. 1:**
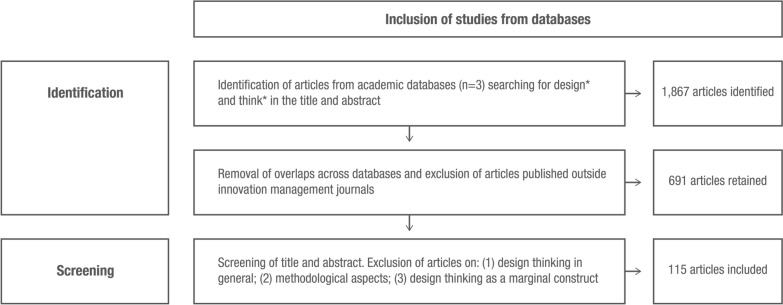
Process used to derive the final sample of 115 articles analyzed within the problematization method

To systematically search for literature in this space, we departed from a recent article published by Micheli et al. (2019) as a blueprint. To this end, we applied “Design*” and “Think*” as search strings within the title or abstract of peer reviewed journals across three databases (ProQuest, Scopus, Science Direct) over the period 2006–2022. The start date of 2006 was chosen for the search because this is when the term design thinking first emerged in the innovation management discourse within an interview article (Dunne & Martin, 2006), followed by two seminal publications (Brown, 2008; Martin, 2009). Our search returned 11,867 articles. To ensure a focus on innovation management literature, we retained articles published in management journals recognized as core by the innovation management research community (as listed by Thongpapanl, 2012). We also retained articles from some additional management journals included in the *Association of Business School Academic Journal Quality Guide* and emerged as key outlets for innovation management research in more recent years (Micheli et al., 2019). We also included articles published in two journals at the crossroad between design and innovation management (i.e., *Design Management Journal* and *Design Management Review*). We removed articles appearing in more than one database, ending up with 691 articles. Subsequently, two of the authors scanned and discussed the title and abstracts of the articles and eliminated those not focusing specifically on design thinking as an approach for innovation management. Particularly, we excluded articles discussing design thinking in general, or focusing on methodological aspects of design thinking, or including design thinking as a marginal concept. This step resulted in a final sample of 115 articles published in 42 journals. Figure 1 visualizes the process to derive this final sample.

The second step of problematization was to analyze all the papers in the sample to uncover their shared underlying assumption. As an essential part of the broader problematization process, this analysis was based upon the principles of critically reviewing the extant literature as described by Alvesson and Sandberg (2020). In particular, the analysis was performed with a reflective approach, avoiding taking consolidated views for granted, while maintaining a selective focus on a specific aspect of the literature. Accordingly, the lead researcher made a full-text reading of all articles in the sample, with a focus on identifying all mentions of the economic, social, and/or environmental impacts of applying a design thinking approach in innovation management, in line with sustainable business theory (Elkington, 1998). This focus was informed by sustainability theory (Elkington, 1998). All relevant passages were highlighted, leading to a preliminary categorization of the articles. To prevent subjective bias and improve reliability of the categorization, two other authors screened the highlighted passages of the articles and accordingly reviewed the preliminary categorization, making comments and changes where appropriate. The lead researcher then used their inputs to make a final categorization, which is reported in Appendix A, and summarized in Table 1.

**Table 1:** Innovation management literature discussing the economic, social, and environmental impact of design thinking

Design thinking articles			
All	Economic impact	Social impact	Environmental impact
Discuss design thinking as an innovation management approach	Discuss design thinking as an approach for businesses to gain competitive advantage through superior innovation performance, elaborating on implementation drivers and barriers	Discuss design thinking as a user-centered approach that businesses, nonprofits, and governments can apply to improve people’s lives through social innovation	Discuss design thinking as an approach to address pressing environmental issues, without elaborating on the nature of such issues, and providing limited evidence from real cases
115 articles	104 articles	46 articles	8 articles
100%	90%	40%	7%

Table 1 shows that all the innovation management articles on design thinking were published between 2006 and 2022. In line with the innovation management view of design thinking, 90% of the articles focus on economic impact and discuss design thinking as an approach for businesses to gain a competitive advantage through superior innovation performance. 40% of the articles focus on social impact and discuss design thinking as a “user-centered” approach that businesses, nonprofits, and governments can apply to improve people’s lives through social innovation. Social impact is thus discussed significantly less than the economic impact. Even more significant is that only 7% of the articles focus on environmental impact. These few articles mention that design thinking can in principle be helpful to address pressing environmental issues. However, this small fraction of research does not elaborate on the nature of such problems and provides only limited empirical evidence on how to resolve them.

We collectively reflected on the results reported in Table 1, aiming to uncover the root causes behind the imbalance of focus between economic, social, and environmental impact. We did so through a series of group and face-to-face discussions within the author team. In addition, we engaged in various informal discussions on the subject with other academics and innovation practitioners in our networks. These discussions led to the insight that a main reason why most articles focus on economic impact at the expense of social and environmental impacts is rooted into an underlying assumption embedded in two seminal texts: “Design Thinking” by Brown (2008), and “The Design of Business: Why Design Thinking is the Next Competitive Advantage” by Roger Martin (2009). The underlying assumption of these texts is:

Design thinking is an approach that supports organizations in gaining competitive advantage, by balancing the desirability, feasibility, and viability criteria in their innovation processes and outcomes.

The third step of problematization was to evaluate this assumption. Desirability is about identifying customers and interpreting what they need and want. Feasibility is about leveraging resources and the stakeholder network to create better innovation outcomes which respond to these wishes. Viability is about finding a profitable and scalable business model to profit from the effort. Balancing these criteria can lead to better innovation outcomes, hence competitive advantage. This assumption emerges rather explicitly from a combined read of the two texts. Most of the articles in our sample consistently cite these texts proposing various and essentially analogous formulations of the same assumption, while largely taking for granted its validity, and considering it as a cornerstone of the consolidating design thinking paradigm (Verganti et al., 2021). In other words, this assumption is closely related to the ontology and fundamental constituents of the design thinking paradigm. Literature on the problematization approach recognizes that such long-standing and explicit assumptions related to paradigms that remain taken-for-granted over time, often present problematic aspects in need of reconsideration (Alvesson & Sandberg, 2013). Our collective reflections and discussions on the subject resonate with this view.

In the awareness of the pressing challenges posed by sustainable development (Brundtland, 1987; ***United Nations, 2015), we argue that the assumption at hand has become problematic, misleading the scope of the design thinking literature. The notion of organizations competing to innovate is not necessarily in contrast with sustainable development. On the contrary, healthy competition can become an engine to solve environmental and social problems (Garriga & Melé, 2004; Waheed & Zhang, 2022). However, when competition is driven only by an economic logic based on limited and / or biased criteria, environmental and social issues are neglected and treated as acceptable externalities, resulting in negative impacts (Elkington, 1998; Johnston et al., 2021). In this regard, the desirability, feasibility, and viability criteria have intrinsic limitations, which render the assumption problematic.

Feasibility and viability relate to technical and financial concerns, while desirability is functional to consider what people need and want. Thus, environmental and social impacts are clearly not yet an integral part of the design thinking equation, which may result in negative impacts in practice. This blind spot is clearly visible in the results of our critical literature review. Concerning social impacts, we note that the desirability criteria present some critical tensions with what may be desirable for society as a whole. What individual consumers want for themselves is intrinsically subject to individual bias and may be in contrast with the collective interest of society (Godelnik, 2017; Hardin, 1968). For example, frequently buying a brand-new smartphone is desirable for many individuals; yet, it also produces a negative impact on the environment (e.g., mining, greenhouse emissions, resource depletion) and on society (e.g., conflict minerals, exploitation of workers) along the supply chain (Akemu et al., 2016). At the same time, whether these individuals truly desire the new phone is debatable as well, because in a free-market economy, firms are incentivized to deliberately create new consumer needs to thrive, often at the expense of human well-being (Bauman, 2013). This example resonates with how desirability is discussed in the innovation management literature on design thinking, which explicitly connects the concept with individual needs of customers and commercial success of firms (Elsbach & Stigliani, 2018, p. 23). This narrative revolving around individual users and customers is likely to result in negative externalities for society. Another example is provided by the unchecked rise and adoption of mobile devices and social networks, which has enabled new forms of human connection, but it has also contributed to severe social problems, including political crises in Europe and the United States, spreading of addiction, depression, and other psychological disorders at scale (Andrew & Baker, 2021; Du, 2021).

As these negative impacts of accelerating innovation (which design thinking may contribute to) on society and the environment are becoming more and more evident, the awareness of citizens and governments is increasing rapidly. Business organizations are increasingly held morally and politically responsible for their impact on society and the environment (Scherer & Palazzo, 2011; Scherer et al., 2016). Considering this responsibility, we turn to responsible innovation, a research stream in the field of business ethics urging academics and managers to consider these impacts and proposing an approach to deal with them (de los Reyes & Scholz, 2022; Gutierrez et al., 2022; Voegtlin & Scherer, 2017). Aiming to explore if responsible innovation ideas can inform the problematic assumption behind the business view on design thinking, in the next section, we make a comparative analysis of both approaches.

## Comparative Analysis of Design Thinking and Responsible Innovation Approaches (Problematization Step 4)

This section covers the fourth step of the problematization method. We propose an alternative assumption by turning to the literature on responsible innovation. We make a comparative analysis of the design thinking and responsible innovation approaches. To do so, we first characterize both approaches in a detailed manner and then discuss similarities and differences.

**Fig. 2:**
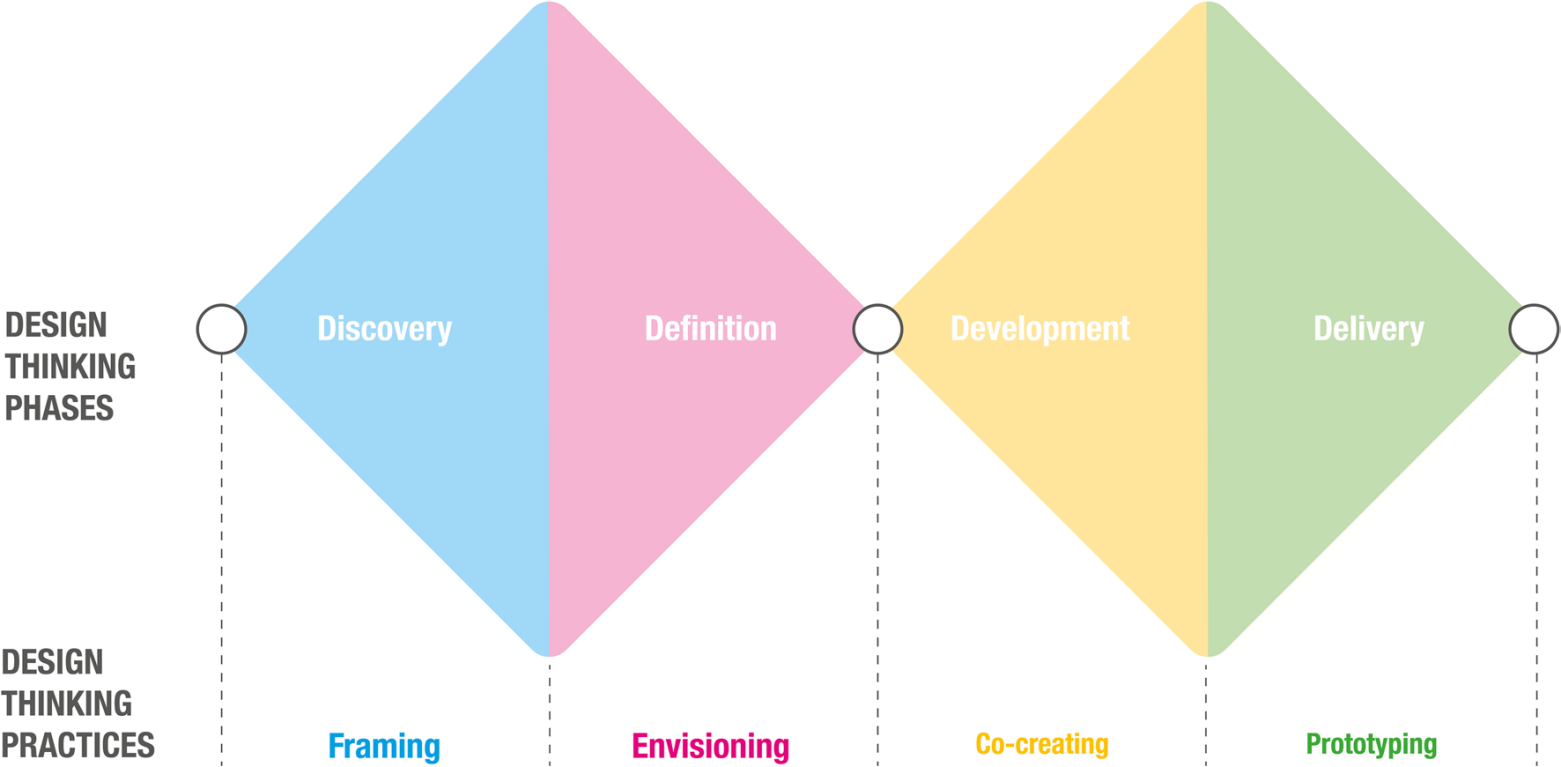
Double Diamond model and underlying practices for “doing design thinking.” Based on: British Design Council (2007), Elsbach and Stigliani (2018) and Gruber et al. (2015)

To describe the design thinking approach, we use the Double Diamond model from the British Design Council (2007). This model describes the design thinking approach as an iterative process unfolding through a sequence of four phases. We selected this model based on a consultation with experts, because it is also often used in the articles included in our critical review (e.g., Elsbach & Stigliani, 2018; Gruber et al., 2015). Then, we clustered the practices within each step and focused on the most frequently mentioned ones. To validate our selection, we again consulted academic and professional experts, who helped us in addressing open issues (e.g., where to place practices that are strongly linked to more than one phase of the design thinking process). Figure 2 specifies the phases and the selected practices. The process starts with a *discovery phase* (visualized in blue). In this phase, a problematic situation is explored by identifying its boundaries, stakeholders, and constraints. The goal is to generate a point of view (i.e., the practice of framing) to inform innovative solutions (Hey et al., 2007; Schön, 1988). In the subsequent *definition phase* (visualized in pink), the different perspectives and insights related to the problem space are integrated into a shared vision (i.e., the practice of envisioning) and objectives for the innovation process (Fuller, 1957; Verganti, 2008). During the *development phase*, (visualized in yellow) involved stakeholders collaboratively conceptualize and discuss potential ideas (i.e., the practice of co-creating) to shape a solution space in which the problem should be addressed (Gemser & Perks, 2015; Sanders & Stappers, 2008). The process concludes with the *delivery phase* (visualized in green), in which solution concepts are quickly built and tested with users and stakeholders (e.g., the practice of prototyping) and then iteratively refined toward implementation (Liedtka, 2015; Schön, 1992).

**Fig. 3:**
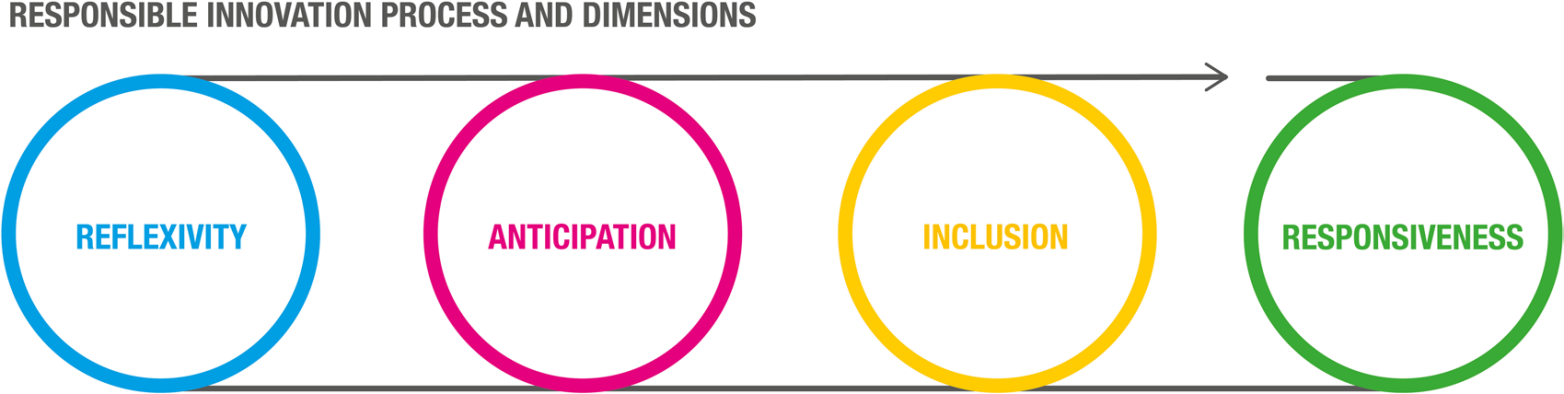
Conceptual representation of responsible innovation. Based on: Stilgoe et al. (2013), Voegtlin and Scherer (2017) and Von Schomberg (2011)

Turning to responsible innovation, on a conceptual level, the scope relates to addressing wicked problems as well, which are linked to global environmental and social issues such as climate change, resource depletion, poverty, and injustice, against the background of economic as well as ethical considerations (Ferraro et al., 2015; Voegtlin & Scherer, 2017). From a structural standpoint, the responsible innovation approach is based on a collaborative, iterative, and experimental process of adaptive learning (Owen et al., 2021; Robaey & Simons, 2015). Since the outcomes of innovation may be difficult to foresee, but have an immediate impact on society, innovating responsibly should enable learning from mistakes to constantly improve solutions for society (Robaey & Simons, 2015). Aiming to better understand this process, Stilgoe, Macnaghten and Owen (2013) proposed four theoretical dimensions that distinguish it from “non-responsible” innovation: reflexivity, anticipation, inclusion, and responsiveness. Drawing on these insights, we propose a visual representation of responsible innovation (Fig. 3). The iterative nature of the process is visualized with a looping line, and the four theoretical dimensions with different colors. Reflexivity (visualized in blue) entails using dialogue to go beyond individual perspectives and jointly reflect on critical issues, and their ethical, social, and environmental implications. Anticipation (visualized in pink) entails thinking in a systemic way and foreseeing plausible and desirable outcomes for innovation. Inclusion (visualized in yellow) entails involving a broader range of relevant stakeholders and collectively negotiating the objective of innovation while taking all interests into account. Finally, responsiveness (visualized in green) entails considering emerging knowledge and insights and consequently adjusting the shape and direction of innovation.

Based on the insights presented in the previous paragraphs, we now compare the responsible innovation and design thinking approaches. Comparing design thinking with other innovation approaches has already been used to provide more solid theoretical grounding to design thinking research and to address its emerging theoretical gaps. For example, Magistretti et al. (2021) recently paralleled design thinking with dynamic capabilities, while Klenner et al., (2021) explored the relationship between design thinking and effectuation approaches, going back to their roots and identifying similarities between their practices and principles. In a similar way, we now compare design thinking and responsible innovation.

Both approaches propose an iterative and experimental business innovation process for addressing wicked problems (Blok & Lemmens, 2015; Plattner et al., 2009; Simon, 1968; Von Schomberg, 2011). Thus, we conclude that at a high level, the design thinking and responsible innovation approaches present interesting similarities in terms of structural features (i.e., iterative and experimental process) and, at least in principle, also in terms of scope (i.e., addressing wicked problems). However, with regard to the scope, detachment arises when moving from the abstract to a more concrete level. Innovation management scholars focus primarily on how design thinking can be leveraged to solve user/consumer problems faster and more effectively, to gain competitive advantage and economic growth (Elsbach & Stigliani, 2018; Micheli et al., 2018). On the other hand, business ethics scholars adopt a holistic focus beyond economic aspects and individual-user needs, elaborating on how an organization, or multiple collaborating organizations, can innovate responsibly in view of wider implications for society and the environment (Ferraro et al., 2015; Voegtlin & Scherer, 2017). Considering the structural similarities and scope detachment, we propose integration of the two approaches, responding to former requests to infuse ethical considerations into design thinking (Greenwood & Freeman, 2018; Hamington, 2019). On these grounds, we propose a new assumption for innovation management research on a more responsible design thinking paradigm. That is:

Design thinking is an approach that supports organizations in collaboratively addressing the challenges of sustainable development, by considering the desirability, feasibility and viability criteria, within responsible innovation processes and outcomes that are ethically acceptable for society and the environment.

This alternative assumption entails going beyond the notion of competitive advantage (i.e., current assumption), considering what people need and want, what is technically possible and what is financially possible, with what is ethically acceptable for society and the environment (i.e., responsibility). Aiming to relate the new assumption to our audience of innovation management scholars, in the next section, we integrate insights from the design thinking and responsible innovation literatures into a conceptual framework.

## Framework for Responsible Design Thinking (Problematization Step 5)

This section covers the fifth step of the problematization method. We relate the alternative assumption to its audience of innovation management and business ethics scholars by advancing a conceptual framework for responsible design thinking. Specifically, linking design thinking and responsible innovation in an integrative framework offers more of an ethical and sustainable grounding for innovation management. The development of the framework is based on a process of conceptual integration (MacInnis, 2011). MacInnis (2011) explains that integration serves to derive conceptual contributions in innovation management research, and accordingly defines it as: “seeing previously distinct pieces as a unified whole whose meaning is different from its constituent parts” (p. 138). In terms of application, MacInnis (2011) explains that “integration involves synthesis of previous findings to generate novel perspectives and frameworks” (p. 146).

**Fig. 4:**
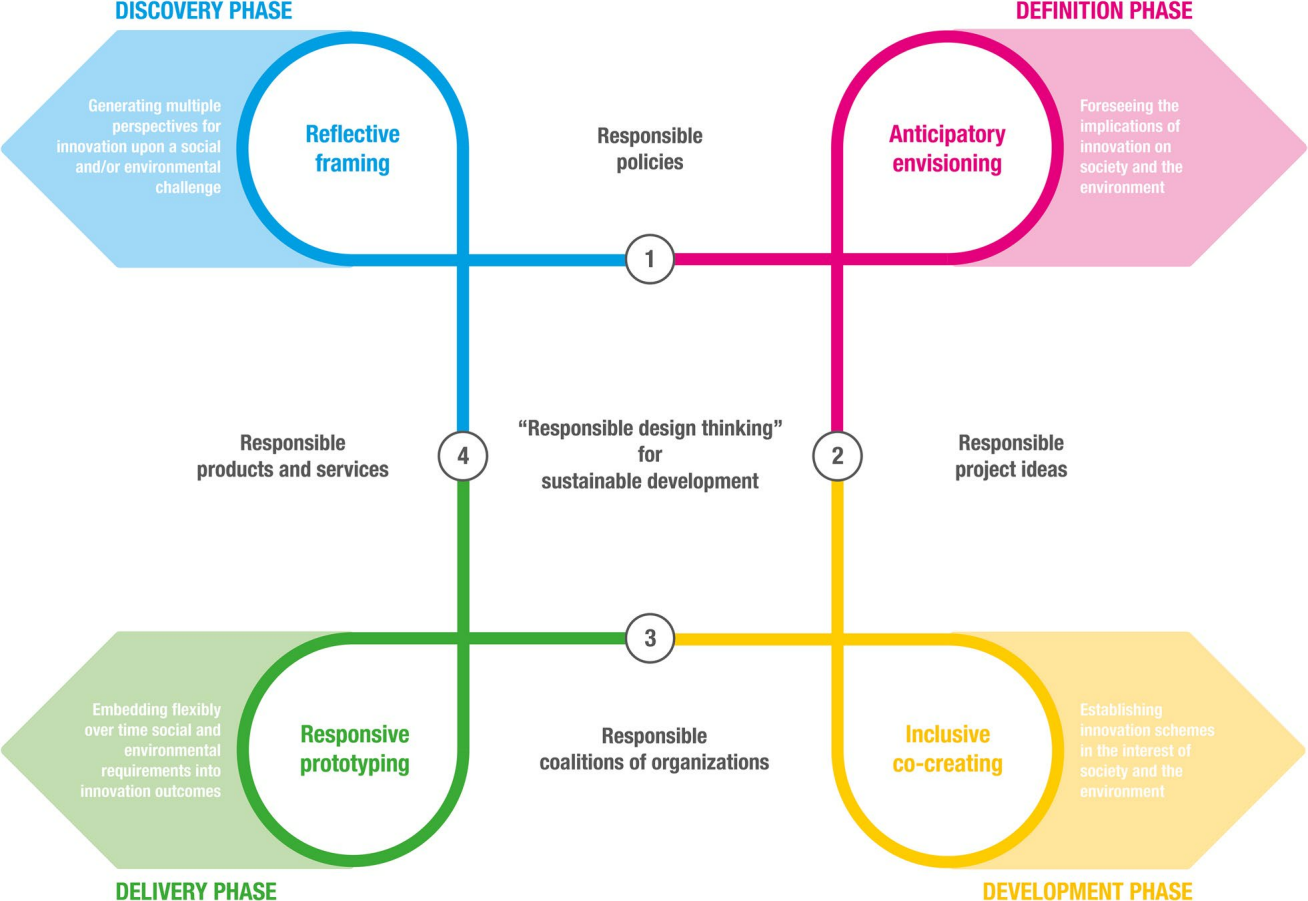
Conceptual framework for responsible design thinking. Based on: British Design Council (2007), Gruber et al. (2015) and Stilgoe et al. (2013)

To accomplish the integration, we first searched the extant literature discussing design thinking practices in relation to the theoretical dimension of responsible innovation. A first selection of texts, including scientific articles and books published in different domains, was done by the authors, based on their knowledge. The sample was enlarged using the reference lists of these texts with a snowballing technique (Wohlin, 2014). Additional literature was also found on Web of Science and Scopus using different combinations of design thinking practices and theoretical dimensions of responsible innovation as keywords. When we identified a potentially relevant text, we did a preliminary screening looking for mentions of a design thinking practice in relation to a responsible innovation dimension, or vice versa. The text was retained only in case this relation was clear and explicit. The process was iterated until we derived enough articles to integrate relevant knowledge fragmented across different domains. We did a full-text screening of the contributions looking for passages discussing how a specific design thinking practice may be linked with a theoretical dimension of responsible innovation, and what the outcome of such a link may be. The one-to-one link between design thinking practices and responsible innovation dimensions gradually emerged and the related findings were synthesized into four tables, which are reported in Appendix B. These tables represent the foundations of our framework, described in the main text of the paper, and are visually synthesized in Fig. 4.

Figure 4 is based on the same color scheme from Figs. 2 and 3. This figure merges the four theoretical dimensions of responsible innovation (i.e., reflexivity, anticipation, inclusion, responsiveness) with the four phases (i.e., discovery, definition, development, delivery) and four practices (i.e., framing, envisioning, co-creating, prototyping) of design thinking.

Responsible design thinking starts with a discovery phase (blue), to generate multiple perspectives for innovation around a social and/or environmental challenge (i.e., reflective framing), turning the challenge into responsible policies (number 1). Consequently, in the definition phase (pink), the implications of innovation on society and the environment are foreseen (i.e., anticipatory envisioning), turning the policies into specific responsible project ideas (number 2). Then, the development phase (yellow) establishes concrete innovation schemes in the interest of society and the environment (i.e., inclusive co-creating), resulting in responsible coalitions of organizations (number 3). Finally, the delivery phase (green) embeds social and environmental requirements flexibly over time into innovation outcomes (i.e., responsive prototyping), leading to responsible products and services (number 4). While the outcome of innovation cannot be fully foreseen, its consequences may have an immediate impact on society and the environment (Robaey & Simons, 2015). Hence, emerging products and services do not represent the end of the process: they shape and modify the original challenge, resulting in a new cycle of a continuous effort and reflection. Furthermore, the process is rarely characterized by a linear sequence (British Design Council, 2007), as indicated by the four loops featured in each of the four phases. The next paragraphs will discuss the theoretical connections underlying the core elements of our conceptual framework. Overall, and in contrast with common design thinking frameworks (e.g., the Double Diamond), its novelty lies into outlining design thinking practices in new distinctive ways, which serve to achieve responsible innovation outcomes and address sustainable development challenges. This was possible thanks to the integration of performative design thinking knowledge with theoretical responsible innovation insights. Appendix B provides supplementary evidence in this regard.

### Discovery Phase: Framing, Reflexivity, and Responsible Policies

The *discovery phase* serves to identify a problematic situation, including its boundaries, opportunities and constraints, as well as generating a problem space (Gruber et al., 2015). In this phase, the design thinking practice of framing may be leveraged to realize the responsible innovation dimension of reflexivity. The design thinking literature explains that framing entails asking open, hypothetical, and provocative questions to challenge existing beliefs and iteratively determine a point of view that allows interpreting and solving the problematic situation (Paton & Dorst, 2011; Schön, 1988). Thus, framing allows (re)interpreting problems to lay the foundation for the design of innovative solutions (Dorst, 2011), iteratively transforming an abstract reflection into the premise for concrete action (Schön, 1983). In parallel, the responsible innovation literature explains that reflexivity requires using dialogue to go beyond individual perspectives and jointly reflect on a sustainable development challenge (Stilgoe et al., 2013; Voegtlin & Scherer, 2017). Former literature has already linked framing and reflexivity. For example, Schön and Rein (1994) argued to leverage framing in order to make sense of the “intractable controversies” of society and create new policy frames for addressing them in a more informed manner. Building on these grounds, we posit that in the discovery phase of the design thinking process, the iterative practice of framing contributes to reflecting deeply and jointly on a sustainable development challenge and turning it into a problem space. Former research indicates that this type of problem space may become concrete through responsible policies, which represent the emergent foundation for public debate and consequent action. Thus, it is important to clarify that the responsible design thinking practice of reflective framing is not about converging toward the definition of a single point of view to redefine an innovation project brief—like “regular” framing (e.g., Gruber et al., 2015)—but rather about a divergent discovery effort for generating multiple and diverse perspectives to foster debate on complex issues affecting the communal good (Vliegenthart & Roggeband, 2007; Windahl et al., 2020). The definition of circular economy policies and calls for industry actions represent s concrete examples of reflective framing, where multiple stakeholders gather in technical working groups to deliberate on the boundaries and cornerstones of a solution space for potential responsible innovation efforts addressing waste management and resource scarcity issues. This example is pertinent, since the circular economy and responsible innovation constructs are linked to each other in EU policy making, within the Horizon 2020 Framework Programme (Burget et al., 2017; European Commission, 2018, 2019a, 2019b; Hartley et al., 2023).

### Definition Phase: Envisioning, Anticipation, and Responsible Project Ideas

In the *definition phase*, insights combine into a problem definition to aid in generating meaningful solutions for the problem at hand (Gruber et al., 2015). The design thinking practice of envisioning may feature in this phase to realize the responsible innovation dimension of anticipation. The design thinking literature explains that envisioning entails gradually and iteratively defining future directions and using these future directions as starting points for defining innovative solutions (Fuller, 1957; Karpen et al., 2017). Envisioning is crucial for design-driven innovation (Verganti, 2008) to conceive new meanings for product and service solutions, and thus pursue radical innovation directions. In parallel, the responsible innovation literature explains that anticipation refers to thinking systemically about a sustainable development challenge, foreseeing innovation outcomes that are socially desirable, environmentally reasonable, and ethically acceptable (Stilgoe et al., 2013; Voegltin and Scherer, 2017). Earlier literature already linked envisioning and anticipation. Six decades ago, Buckminster Fuller (1957) advanced the idea of a “comprehensive anticipatory design science” to define a “global vision” to guide responsible human development toward a desirable future, addressing sustainability problems by “planning innovation” projects. More recently, Prestes Joly et al. (2019) argued that envisioning new service ecosystem projects is essential to enable institutional change, and ultimately anticipate a better future based on more sustainable lifestyles and consumption practices. On these grounds, we posit that in the definition phase of the design thinking process, the iterative practice of envisioning serves to anticipate specific objectives for a broader sustainable development challenge. Former research indicates that these objectives may be embedded into responsible project ideas. Thus, the responsible design thinking practice of anticipatory envisioning is not about developing future scenarios of how users will interact with new design concepts—like “regular” envisioning (Plattner et al., 2009)—but rather striving to foresee the implications of innovations on human development and livability on the planet (Fuller, 1957; Rockström et al., 2009). Going back to the former circular economy example, after a solution space for interventions is defined through reflective framing, the definition of specific project ideas, complemented by a prospective estimation of foreseen environmental (e.g., carbon emission reduction), social and economic impacts (e.g., employment creation), represents an anticipatory envisioning effort.

### Development Phase: Co-Creating, Inclusion, and Responsible Coalitions of Organizations

The *development phase* revolves around collaborative and creative approaches to engage project stakeholders in developing a solution space (Gruber et al., 2015). In this phase, the design thinking practice of co-creating may contribute to realizing the responsible innovation dimension of inclusion. The design thinking literature explains that co-creating entails collaborative and iterative generation of innovation solutions by leveraging inputs and resources from the involved stakeholders (Gemser & Perks, 2015; Micheli et al., 2018). To this end, a variety of tools have been developed, including Lego Serious Play and rapid co-creation, as well as a variety of brainstorming techniques (Bocken et al., 2023; Brown, 2008; Gardien et al., 2016; Roos et al., 2004). In parallel, the responsible innovation literature uses the conceptual dimension of inclusion to indicate the importance of working collaboratively while addressing the complexity of sustainable development challenges (Mazzucato, 2018; Stilgoe et al., 2013). Earlier literature has linked co-creating and inclusion. For example, Voegtlin and Scherer (2017) discuss “inclusion” in terms of global solutions for sustainable development co-created by multiple firms, NGOs and governments. Manzini (2017) links the responsibility of co-creating a more cohesive and resilient society with the establishment of new coalitions of organizations. Building upon this line of argumentation, we posit that in the development phase of the design thinking process, the iterative practice of co-creating ensures the inclusion of multiple stakeholders in a solution space. Former research indicates that this solution space may emerge through the formation of responsible coalitions of organizations. Therefore, the responsible design thinking practice of inclusive co-creating is not simply about the involvement of users and customers—like “regular” co-creating (Gemser & Perks, 2015; Sanders & Stappers, 2008)—but rather about establishing governance schemes and alliances for sustainable development (Voegtlin & Scherer, 2017). Inclusive co-creation is exemplified again in the EU context by the establishment of consortia and alliances including the public sector, academic institutions, business actors, and representatives of local communities, joining forces to cohesively develop solutions for circular industry at a national and regional scale (Baldassarre & Calabretta, 2023; Baldassarre & Saveyn, 2023).

### Delivery Phase: Prototyping, Responsiveness, and Responsible Products and Services

The *delivery phase* centers on iteratively testing promising solutions with stakeholders to gradually adjust, improve, and combine them into a feasible and viable outcome (Gruber et al., 2015). In this phase, the design thinking practice of prototyping helps to accomplish the responsible innovation dimension of responsiveness. The design thinking literature explains that prototyping essentially consists of building an artifact that can be readily implemented and progressively adjusted over time as new requirements emerge (Liedtka, 2015; Schön, 1992). By combining different communication languages (e.g., visualizations, working models), prototypes make intangible business ideas explicit and understandable (Karpen et al., 2017; Kumar & Holloway, 2009). In parallel, the responsible innovation literature explains that responsiveness requires flexibility as to the shape and direction of innovation in reaction to emerging knowledge and to the dynamic and "wicked" nature of sustainable development challenges (George et al., 2016; Stilgoe et al., 2013). Former literature has linked prototyping and responsiveness. For example, Viswanathan and Sridharan (2012) explore how firms can prototype and test product and service concepts in emerging markets, to allow customization and respond to individual and communal needs in a more flexible way. In a similar vein, Baldassarre et al., (2020a, 2020b) and Lubberink et al. (2017) argue that prototyping is an essential practice to plan and execute small-scale pilots, to flexibly experiment with the integration of environmental requirements into the product and service development process, and to respond to sustainability challenges. On these grounds, we posit that in the delivery phase of the design thinking process, the iterative practice of prototyping helps respond to a sustainable development challenge through specific outcomes. Former research indicates that such outcomes may be represented by responsible products and services. Given the complexity of the sustainable development challenges and the number of stakeholders involved, the responsible design thinking practice of responsive prototyping is not a short sprint—as sometimes suggested for “regular” prototyping (Knapp et al., 2016)—but rather a long marathon in which social and environmental criteria are quickly and flexibly incorporated over time into product and service outcomes (Hillgren et al., 2011). A concrete example of responsive prototyping is provided by a recent Horizon 2020 circular economy project (zerobrine.eu), where collaborating organizations progressively built with a trial-and-error approach over the course of four years, a demo-plant in the Port of Rotterdam, to recover high-purity minerals from industrial wastewater, improving waste management and resource efficiency at a local scale.

## Discussion and Research Agenda (Problematization Step 6)

This section covers the sixth step of the problematization method, which reflects upon the alternative assumption and framework to guide future research. The reflection is informed by a series of brainstorming sessions, in which the first three authors discussed the contribution of this work, as well as in a workshop with external academic experts in the innovation management community. The aim is moving innovation management research forward by integrating it with business ethics literature, proposing relevant questions within a research agenda for responsible design thinking.

The starting point of the reflection is the critical literature review and identification of a core assumption underlying innovation management research on design thinking. That is: design thinking is an approach that supports organizations in gaining competitive advantage, by balancing the desirability, feasibility, and viability criteria in their innovation processes and outcomes. This assumption relates to the ontology and fundamental relevance of the design thinking paradigm and has been taken for granted in the innovation management literature (Calabretta et al., 2016, 2017). Nevertheless, the assumption is problematic because the desirability, feasibility, and viability criteria have intrinsic limitations that put emphasis on economic growth, while neglecting the social and environmental problems seen as externalities, while governments and citizens increasingly hold organizations morally and politically responsible for these impacts (Scherer & Palazzo, 2011; Scherer et al., 2016). Consequently, we propose the alternative assumption that design thinking is an approach that supports organizations in collaboratively addressing the challenges of sustainable development, by considering the desirability, feasibility and viability criteria, within responsible innovation processes and outcomes that are ethically acceptable for society and the environment. This alternative assumption flows into our conceptual framework that specifies four responsible design thinking practices: reflective framing, anticipatory envisioning, inclusive co-creating, and responsive prototyping. In contrast with common design thinking frameworks, these practices are now adapted to the specific purpose of responsible innovation. The proposed responsible innovation practices represent mechanisms to concretely design responsible outcomes toward sustainable development, by gradually unpacking and addressing the epistemic and moral barriers characterizing environmental and social wicked problems (Blok & Lemmens, 2015; de Hoop et al., 2016). These wicked problems, such as climate change and poverty, often manifest themselves only partially, impeding full understanding of all their implications (i.e., epistemic barriers). Moreover, they have no “right-or-wrong” solution, and how they are addressed depends upon subjective interpretations and arbitrary choices (i.e., moral barriers). For example, biofuels may represent a responsible alternative to fossil fuels to lower greenhouse emissions causing climate change. However, intensive production of biofuels results in other environmental and social issues including biodiversity loss, unjust appropriation of land in developing countries, and food-production issues, leading to an international conflict of interest in which the different parties involved have significantly different decision-making power (Blok & Lemmens, 2015). In this context, doing innovation without a sense of ethics may not lead to desirable outcomes for humanity, hence the relevance of responsible design thinking practices in ensuring business sustainability. For instance, inclusive co-creation can be a way to collectively discuss concrete options among all the aforementioned parties—not only governments, innovators, and consumers in the rich world, as it often happens with common co-creation practices (Baldassarre & Micciché, 2014; Gemser & Perks, 2015)—in order to generate a coalition of organizations focusing on biofuel production by taking into account the needs of industrialized countries, as well as those of developing ones. Taking a few steps back in the process, reflective framing—which is more about “public debate” in comparison with how framing is commonly applied within the design thinking process (Vliegenthart & Roggeband, 2007)—may allow for political deliberation, resulting in responsible policies to mitigate the private business interest of individual organizations by, for instance, broadening the problem space from biofuel production, to renewable energy production, critically evaluating pros and cons of other alternatives, such as hydrogen production or photovoltaic panels (Axt et al., 2023; Baldassarre et al., 2023; Nyffenegger et al., 2023).

**Fig. 5:**
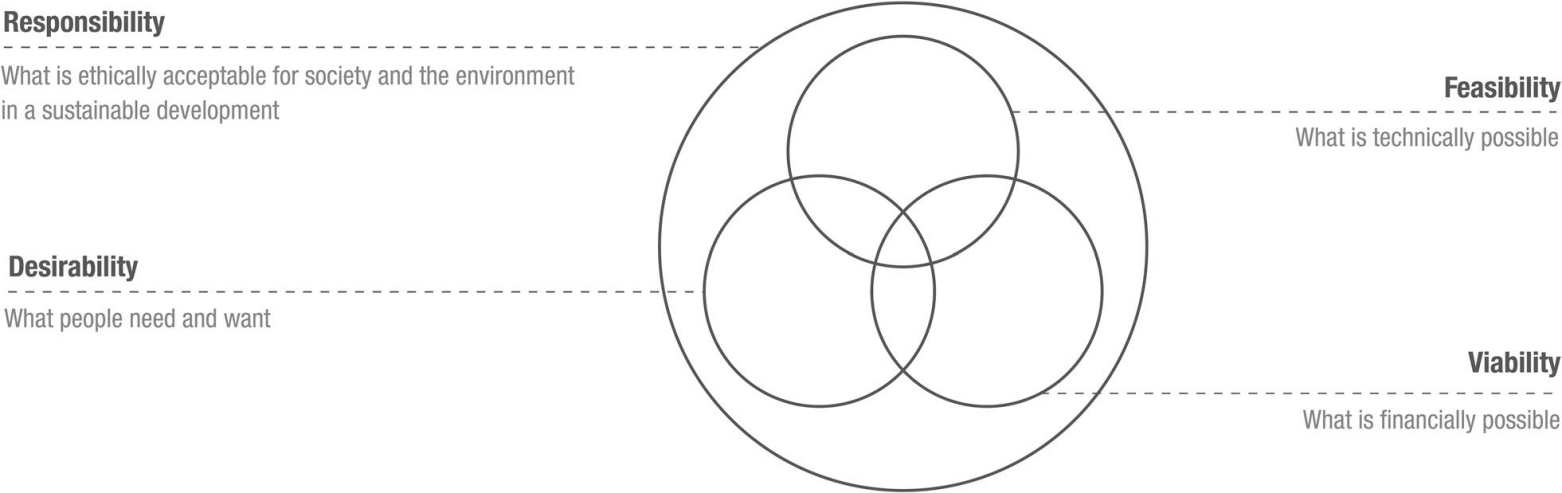
Desirability, feasibility and viability criteria of design thinking, grounded in the responsibility criteria. Based on: Brown (2009) and the outcomes of this study

The main contribution of this article consists of linking design thinking practices to responsible innovation dimensions. This link is important for two reasons. First, design thinking literature has been repeatedly criticized by innovation management scholars for lacking theoretical grounding (Dell’ Era et al., 2020; Johansson-Sköldberg et al., 2013). As a result, a recent review argues that the design thinking discourse has unfolded “in a vacuum” (Verganti et al., 2021, p. 163). Other recent literature suggests that connecting to other innovation theories is essential to strengthen the theoretical underpinnings of design thinking (Gemser & Barczak, 2020; Klenner et al., 2021; Magistretti et al., 2021). The second reason is that the design thinking literature has so far failed to sufficiently account for ethical questions related to social and environmental impacts when applying the underlying approach in business innovation, as demonstrated by the results of our systematically conducted critical review. The results resonate with a recent call to action: Hamington (2019) explicitly argues for the need for research integrating business ethics into design thinking literature. In combination, these two reasons demonstrate the value of linking design thinking’s performative knowledge around specific business innovation practices (Gruber et al., 2015), with responsible innovation’s conceptual knowledge around theoretical dimensions, which distinguish responsible from “non-responsible” innovation efforts (Stilgoe et al., 2013). Thus, the contribution expands the understanding of design thinking practices beyond actions and interaction patterns to achieve superior innovation outcomes driven by private business interest and a competitive advantage logic (Martin, 2009; Reckwitz, 2002), openly acknowledging their value for innovating responsibly driven by collective concerns (Porter & Kramer, 2011; Voegtlin & Scherer, 2017). This also realigns the ongoing “ahistorical and apolitical” (Staton et al., 2016, p. 2) innovation management discourse on design thinking with its original design science roots, which called for the responsibility of designers and innovators to deal with pressing environmental and social issues (Fuller, 1957, 1969; Papanek, 1971)—rather than narrowly supporting companies in gaining competitive advantage (Martin, 2009, 2010). On these grounds, building on our proposed alternative assumption, and differently from other design thinking frameworks, we recommend explicitly reintroducing “responsibility” (i.e., what is ethically acceptable for society and the environment in a sustainable development) as the overarching effectiveness criterion of design thinking, beyond those of desirability (i.e., what people need and want), feasibility (i.e., what is technically possible), and viability (i.e., what is financially possible), as visualized in Fig. 5.

Grounding the desirability, feasibility, and viability criteria into responsibility entails a clear organizational purpose, cross-organizational collaboration, and a focus on reconciling the tensions that naturally emerge in sustainable innovation processes between economic, social, and environmental impacts (Hahn et al., 2015; Mazzucato, 2018). The smartphone example is again appropriate to illustrate these tensions and how they may be reconciled by applying the responsible design framework in practice. Within an orthodox design thinking logic, consumer electronics companies such as Apple and Samsung are used by scholars as case studies on how desirability, feasibility, and viability are balanced successfully, disregarding the environmental and social impacts of their activities (Chang and Joo, 2013). On the other hand, companies such as Fairphone, operating in line with a responsible design thinking paradigm, strive to develop a product that is feasible, viable and desirable within an ethical framework where human rights and resource depletion are not treated as externalities, but become cornerstones in the innovation process (Akemu et al., 2016). The Fairphone case shows that the tensions between feasibility, viability and responsibility can indeed be managed by designing a fair value chain and developing an upgradable and durable product design. At the same time, the problematic tensions between desirability and responsibility can be managed by creating a brand that nudges consumers to reconsider what they need and want for themselves, in view of a greater social good, encouraging and empowering to choose a product that does not support unsustainable mining practices and can be repaired to reduce pressure on resource extraction.

Ultimately, we see this conceptual grounding of the popularized design thinking criteria into responsibility as a way to stimulate innovation management scholars to engage in a critical reflection on the subject (Hamington, 2019). Besides the argument of competitive advantage, the rhetoric of design thinking and the related practices has focused on a “user-centered” approach that can be used for social innovation. However, there is little critical discussion around its shortcomings and how the methodology will need to be adapted to “do good.” In this regard, Staton and colleagues (2016) recently warned that “mainstream design thinking methodologies are limited by their myopic focus on technological innovation” (p. 1), and that “there are ironies around the use of design thinking for social impact in its contemporary market-friendly iterations” (p. 2). In a similar vein, Vink and Koskela-Huotari (2022) explain that “the mere adoption of (design) methods, without understanding the underlying principles (such as ethical or sustainable considerations) that guide their use, cannot produce the transformative outcomes for which they were developed initially” (p. 1). Indeed, attempts of adapting the approach to primarily address social and environmental challenges are now emerging both in research and in practice, as also demonstrated by a recent report of the British Design Council (2021) about design thinking beyond “net-zero” impact. Acknowledging the importance of these efforts and aiming to catalyze them, we derived a framework integrating ethical and sustainable considerations into specific design thinking practices.

The responsible design thinking framework may support future practice by supporting sustainable design and management efforts as well as other key decision-making areas as diverse as urban planning and policymaking, where design thinking is starting to be used (Johansson-Sköldberg et al., 2013; McGann et al., 2018; Mintrom et al., 2016; Müller-Seitz & Weiss, 2021). Because design thinking is an approach used increasingly across different actors for complex decision-making, a framework that embodies responsible innovation and environmental and social issues is essential to avoid over-simplistic conclusions and solutions. For example, McGann et al. (2018) question whether an innovation lab approach using design thinking is the best place to fix ‘complex issues’ such as policy making. In particular the user-centered design-led labs often used for policymaking (as opposed to e.g., evidence-based labs) (McGann et al., 2018), may benefit from a responsible innovation approach because they would then more prominently focus on social and environmental problems in addition to immediate user concerns. Furthermore, as such design-led labs focus on generating and testing ideas rather than implementing and scaling them (McGann et al., 2018), a more reflexive, anticipatory, inclusive, and responsive approach to design thinking would also potentially lead to better long-term and scalable solutions that benefit the society and natural environment rather than short-term limited stakeholder concerns. With a responsible design thinking framework as the new standard of innovation practice, design thinking has the potential to move from an engaging and creative concept toward a more profound approach to help resolve social and environmental problems.

On the theory side, to advance future research upon the framework, we now propose a research agenda for responsible design thinking. Using our framework as a starting point, we propose two main directions for future research. The first direction calls for more research “looking within the framework,” while the second direction aims to catalyze efforts “looking beyond the framework.” For each direction, we pinpoint several potential questions, as specified in Table 2, and further discussed in the following paragraphs.

**Table 2:** Future research directions and questions for responsible design thinking

Future research on responsible design thinking	
Looking within the proposed framework	Looking beyond the proposed framework
How can innovators perform reflective framing to take responsibility for the impact of innovation on the economy, society and the environment?	What are the human conditions (micro level) needed to innovate through responsible design thinking?
How can innovators perform anticipatory envisioning to take responsibility for the impact of innovation on the economy, society and the environment?	What are the organizational conditions (meso level) needed to innovate through responsible design thinking?
How can innovators perform inclusive co-creating to take responsibility for the impact of innovation on the economy, society and the environment?	Which economic sectors, environmental and social challenges (macro level) are most suitable for, or in need of, responsible design thinking?
How can innovators perform responsive prototyping to take responsibility for the impact of innovation on the economy, society and the environment?	How does responsible design thinking align and / or differ from other ethical innovation approaches and theories (meta level)?

### First Future Research Direction: Looking Within the Responsible Design Thinking Framework

The first research direction is about looking at the constituting elements inside the framework, to better understand the real impact of responsible design thinking practices. The present article links design thinking practices and responsible innovation dimensions on a conceptual level. We label four responsible design thinking practices, namely reflective framing, anticipatory envisioning, inclusive co-creating, and responsive prototyping. We also indicate, again on a conceptual level, what may be the outcomes of performing these practices, namely responsible policies, responsible project ideas, responsible coalitions of organizations, responsible products, and services. In addition, we provide concrete examples about the practices and outcomes. However, more research is needed to fully understand how the practices are performed in business innovation, to take responsibility for its impacts on the economy, society and the environment. To this end, we put forward four questions for future research, which zoom into four specific areas of the proposed responsible design thinking framework.

#### How can Innovators Perform Reflective Framing to Take Responsibility for the Impact of Innovation on the Economy, Society, and the Environment?

Future research could further investigate how the design thinking practice of framing can be applied to operationalize reflexivity in responsible innovation by supporting stakeholders in the discovery and integration of diverse perspectives into responsible policies (Stilgoe et al., 2013; Vliegenthart & Roggeband, 2007; Windahl et al., 2020). Recent research by Vink and Koskela-Huotari (2022) points to the need for and benefits of greater reflexivity in using design approaches, while arguing that empirical demonstrations remain scant.

#### How can Innovators Perform Anticipatory Envisioning to Take Responsibility for the Impact of Innovation on the Economy, Society, and the Environment?

Future research may explore how the practice of envisioning allows to perform anticipation in innovation, by fostering the definition of more systemic future scenarios and related responsible project ideas, bearing in mind their environmental and social consequences (Fuller, 1957; Stilgoe et a., 2013). Previous responsible innovation research has already pointed at methods like technology assessments, horizon scanning, or scenario planning for operationalizing the theoretical dimension of anticipation (Stilgoe et al., 2013). However, the same research also recognizes the limitations of these methods in supporting science and technology stakeholders in transitioning from attempting to predict responsible futures to enacting these futures by mobilizing resources. In this regard, future research may leverage our framework and explore, for instance, how the design practice of envisioning uses imagination to not only describe a desirable future, but also to direct the intentions of participating stakeholders toward that future by establishing plausible directions, intermediate projects, and relevant milestones (Rindova & Martins, 2021). By defining a direction of exploration and development, the envisioning practice reduces stakeholders’ reluctance to anticipate by reducing the perceived distance and uncertainty of the future.

#### How can Innovators Perform Inclusive Co-creating to Take Responsibility for the Impact of Innovation on the Economy, Society, and the Environment?

Future research may investigate how the practice of co-creating concretely achieves inclusion in innovation, by enabling the active involvement of stakeholders in the development of cross-organizational collaborations and new governance schemes that take up the responsibility of solving global problems (Manzini, 2017; Stilgoe et al., 2013; Voegtlin & Scherer, 2017). Empirical research in responsible innovation so far has mainly focused on characterizing the role and interactions of a subset of these stakeholders (i.e., scientific institutions, technology providers, policy makers) (Owen et al., 2021). We suggest that further research needs to investigate whether and how co-creation can be leveraged to overcome the barriers hindering a wider and more active engagement of stakeholders, such as the perceived complexity of responsible innovation goals or the power, political, and institutional (socio-cultural) dynamics occurring within responsible collaborations.

#### How can Innovators Perform Responsive Prototyping to Take Responsibility for the Impact of Innovation on the Economy, Society, and the Environment?

Future research may focus on the practice of prototyping as a mechanism to enact responsiveness in innovation by enabling collaborating stakeholders to flexibly incorporate new requirements over time (Hillgren et al., 2011; Simon, 1995; Stilgoe et al., 2013). Previous innovation research already mentioned prototyping as a practice to pilot first versions of solutions for further improvement (Lubberink et al., 2017), while Mink et al. (2014) and Baldassarre et al., (2020a, 2020b) investigate how prototyping could be applied by innovators for more responsible solutions. However, this research is still at an early stage and empirical evidence is limited, particularly in view of complex ecosystems or networks of actors that are typically involved in responsible innovation projects, working together to deliver often complex and interdependent solutions.

### Second Future Research Direction: Looking Beyond the Responsible Design Thinking Framework

The second research direction is about looking beyond the framework. In this article, we suggest that responsible design thinking is a variant of “regular” design thinking that occurs when the practices of framing, envisioning, co-creating, and prototyping are executed in a specific, more responsible way. For example, the practice of framing becomes responsible when it is reflective, diverging beyond a project brief, to generate multiple and diverse perspectives to foster debate on complex issues affecting the communal good (Vliegenthart & Roggeband, 2007). However, another important area of research relates to the context in which responsible design thinking may occur. More research is needed to better understand this context at a micro, meso, macro, and meta level.

#### What are the Human Conditions (Micro Level) Needed to Innovate Through Responsible Design Thinking?

At a micro level, important questions arise related to the attitudes, character, and capabilities of human beings involved in responsible design thinking. Five decades ago, Victor Papanek (1971) already urged designers to take responsibility for the impact of the innovations they put on the market. Today, this is perhaps even more relevant to deal with increasingly pressing environmental and social challenges (George et al., 2015, 2016). However, limited research is currently performed on who are these “responsible designers”—which Papanek called for. Better understanding their personal motivations and traits, as well as their professional expertise and skills is essential if researchers want to uncover how responsible innovation may be operationalized through design practices (Blok & Lemmens, 2015; Klaassen et al., 2017; Mink et al., 2014). Moreover, understanding who the so-called responsible designers are is also important for educational purposes, to inform universities as they define their academic curricula on design. This kind of research is already present outside the design domain. For example, scholars have analyzed and identified different typologies of sustainable entrepreneurs, distinguishing the social engineer from the social constructionist and the social bricoleur (Zahra et al., 2009). Despite the relevance, similar insights are not yet present when it comes to responsible design thinking. Hence, we encourage future research on the subject.

#### What are the Organizational Conditions (Meso Level) Needed to Innovate Through Responsible Design Thinking?

At a meso level, responsible design thinking entails going beyond typical business considerations (desirability, viability, feasibility), requiring organizations to evolve to uphold their social and environmental responsibility. Recent research on design thinking has already called for an important evolution, arguing that it is now crucial to “elevate the design function to a strategic level in the organization,” to improve innovation performance and outcomes (Micheli et al., 2018, p. 629). One of the core scholarly propositions in this regard is that such elevation may be achieved through certain managerial behaviors (e.g., leadership of the design function, generating awareness, inter-functional coordination), and possibly through the creation of a Chief Design Officer who sits at the top-management table (Micheli et al., 2018; Quint et al., 2022). However, the organizational evolution that is required for responsible design thinking remains to date largely unexplored. What type of leadership is needed? Who can incarnate it, how and why potentially not? These considerations can get quite complex in responsible efforts aiming to address environmental and social challenges, with a large set of stakeholders involved. Relevant research suggests that moving forward requires such stakeholders to work in a more integrated manner, jointly shaping the value creation process, while also reconsidering the notion of value itself (Garriga, 2014). Thus, managers may need to go beyond their typical role behaviors and start working more with other actors outside their organizations, including for example entrepreneurs, policy-makers, and academic institutions. We encourage research on the organizational conditions needed for this to happen more in future.

#### Which Economic Sectors, Environmental and Social Challenges (Macro Level) are Most Suitable for, or in Need of, Responsible Design Thinking?

At a macro level, future research may investigate the possibilities of “doing” responsible design thinking in different contexts. While we adopt the perspective that design thinking should always be applied in a responsible way, we also foresee that doing responsible design thinking may not always be equally easy or suitable in different economic sectors and / or in relation to different social and environmental challenges. When the concept of responsible innovation was introduced as a cross-cutting issue for the Research and Innovation Framework Program Horizon 2020, the European Union also defined specific focus areas to be prioritized, including health, food security, energy, transport, climate, social security, and inclusion (European Parliament, 2018; Von Schomberg, 2013). More recently, the new Framework Program Horizon Europe, in line with the Green Deal, prescribes to focus innovation efforts on the same areas, plus the digital and space industries, as well as on natural resources and raw materials (European Commission, 2019a, 2019b, 2021). In parallel, the United Nations’ agenda remains in place, aiming to address wicked problems, and catalyze the action of organizations around seventeen specific sustainable development goals, such as climate action, but also the elimination of poverty (George et al., 2016; United Nations, 2015). Future research may explore how wicked problem-solving through responsible design thinking can fit best into such agendas. For example, scholars may develop industry categorizations where responsible design thinking is particularly needed or fruitful. Besides the practical relevance of this effort, uncovering a clearer understanding of the contextual fit and conditions for responsible design thinking would contribute to build a stronger theoretical foundation for the approach to move forward.

#### How does Responsible Design Thinking Align and/or Differ from Other Ethical Innovation Approaches And Theories (Meta Level)?

At a meta level, future research on responsible design thinking may seek to connect this framework to other ethical innovation approaches and theories in the literature that are potentially related. For example, connections may be established with literature on sustainable innovation, corporate social responsibility, or circular innovation, to name a few (Baldassarre & Calabretta, 2023; Ciulli et al., 2020; Freudenreich et al., 2020; Garriga & Melé, 2004). Similarly, the design literature comprises areas such as value sensitive design, sustainable design, eco-design, or circular design (Baldassarre et al., 2020a, 2020b; Ceschin & Gaziulusoy, 2016; Friedman & Hendry, 2019). Frequently, however, we observe silo-orientations whereby the various literature streams remain largely independent and disconnected. Recently, Lubberink et al. (2017) argued that this compartmentalization should be overcome, and showed how to do so by comparing “responsible,” “sustainable,” and “social” innovation processes. Similarly, future theorizing could seek to explore what are the conceptual and practical differences between responsible design thinking and alternative approaches and theories.

## Concluding Remarks

While engaging with the content areas in our proposed research agenda, scholars may also attempt to address some of the limitations of our work. Our integration of design thinking practices with responsible innovation dimensions is based primarily upon conceptual argumentation. In this regard, we note that the proposed responsible design thinking process is iterative in nature and relies on a sequence of practices and phases whose boundaries may not always be clear-cut. This aspect is linked to the original conceptualization of the design thinking process in innovation management literature, which partly underlies our framework. Elsbach and Stigliani (2018), as well as Gruber et al., (2015) clarify that throughout the process, a different practice should, from a conceptual standpoint, prevail over the others at different times, depending on the main goal of the phase (i.e., framing in discovery, envisioning in definition, co-creating in development, prototyping in delivery); however, they do not exclude that in reality, more than one practice may come into play at the same time, given the fluid, experimental and iterative nature of design thinking. Therefore, the segregation of phases and practices put forward by our framework should be seen as a conceptual simplification, which is intended to start shedding more clarity on fundamental dynamics that underlie this process but does not fully capture the complexity of reality.

We ultimately do not exclude that different responsible design thinking practices may be performed in parallel by different actors, in the same phase. This limitation related to conceptual simplification might be mitigated by follow-up empirical research investigating how responsible design thinking practices and process would play out in a variety of real cases. This would be highly beneficial for refining the framework as well as for providing actionable guidance to actors willing to use it in practice. Finally, while we discussed our ideas with other academics and business innovation practitioners in our networks, as part of the problematization process, we did not consult with stakeholders from the public sector and policy-making. Further work could take this limitation into account by discussing responsible design thinking with these actors as well, to integrate the whole spectrum of knowledge and expertise that is relevant for responsible design thinking at a macro level.

A final thought that arises when examining the proposed framework and the main contribution of this paper relates to the recent call for consolidating the theoretical foundations of design thinking, through its integration with other innovation theories (Gemser & Barczak, 2020; Verganti et al., 2021). Several scholars are now addressing this call. For example, Magistretti et al. (2021) recently argued that design thinking is a dynamic capability. Similarly, Klenner et al. (2022) highlight connections between design thinking and effectuation theory. In this paper, we also answered the call, arguing that design thinking should be grounded in responsible innovation. However, if different scholars now start consolidating the theoretical foundations of design thinking using a multitude of different innovation theories—which is indeed possible given the flexible and interdisciplinary nature of design as a word and as a discipline (Buchanan, 1992)—their efforts combined may ultimately be partially counterproductive. In other words, while these efforts may help in building a stronger theoretical foundation for design thinking, the plurality of perspectives may also lead to a further conceptual scattering or dilution of design thinking into other theories. There is thus merit in seeking connections (e.g., at micro, meso, or macro level) and coherence (e.g., ontological, epistemological) between these theorizing efforts, perhaps attempting to integrate them going forward. While we indeed intend to work together on this matter with other scholars in this stream of research, we would like to close this article looking backward to inform the future, with a political statement from Victor Papanek (1971) about the role of design “in the real world”: “Design, if it is to be ecologically responsible and socially responsive, must be revolutionary and radical. It must be independent of concern for the gross national product.”

## Supplementary Information

Below is the link to the electronic supplementary material.

Supplementary file1 (DOCX 74 KB)

## Data Availability

Not applicable.

## References

[CR1] Akemu, O., Whiteman, G., & Kennedy, S. (2016). Social enterprise emergence from social movement activism: The Fairphone case. *Journal of Management Studies,**53*(5), 846–877.

[CR2] Alvesson, M., & Sandberg, J. (2011). Generating research questions through problematization. *Academy of Management Review,**36*(2), 247–271.

[CR3] Alvesson, M., & Sandberg, J. (2013). *Constructing research questions: Doing interesting research*. Sage.

[CR4] Alvesson, M., & Sandberg, J. (2020). The problematizing review: A counterpoint to Elsbach and Van Knippenberg’s argument for integrative reviews. *Journal of Management Studies,**57*(6), 1290–1304.

[CR5] Andrew, J., & Baker, M. (2021). The general data protection regulation in the age of surveillance capitalism. *Journal of Business Ethics,**168*(3), 565–578.

[CR6] Archer, B. (1979). Design as a discipline. *Design Studies,**1*(1), 17–20.

[CR7] Axt, M., Baldassarre, B., Kirchherr, J., & Vestergaard, J. (2023). Circular economy in critical value chains: The case of hydrogen electrolysers and fuel cells. In K. Niinimäki & K. Cura (Eds.), *Product lifetimes and the environment 2023—conference proceedings* (pp. 77–83). Aalto University Publication Series. 10.13140/RG.2.2.36270.48961

[CR8] Baldassarre, B. (2021). From problem to solution: A few stories about design and business for sustainable development. Doctoral dissertation. Delft University of Technology.

[CR9] Baldassarre, B., Buesa, A., Albizzati, P., Jakimow-Canton, M., Saveyn, H., & Carrara, S. (2023). *Analysis of Circular Economy Research and Innovation (R&I) intensity for critical products in the supply chains of strategic technologies*. Publications Office of the European Union. 10.2760/582527

[CR10] Baldassarre, B., & Calabretta, G. (2023). Why circular business models fail and what to do about it: A preliminary framework and lessons learned from a case in the European Union (Eu). *Circular Economy and Sustainability*. 10.1007/s43615-023-00279-w

[CR11] Baldassarre, B., Calabretta, G., Bocken, N., Diehl, J. C., & Keskin, D. (2019). The evolution of the strategic role of designers for sustainable development. *Academy for Design Innovation Management,**2*(1), 807–821.

[CR12] Baldassarre, B., Keskin, D., Diehl, J. C., Bocken, N., & Calabretta, G. (2020a). Implementing sustainable design theory in business practice: A call to action. *Journal of Cleaner Production,**273*, 123113.

[CR13] Baldassarre, B., Konietzko, J., Brown, P., Calabretta, G., Bocken, N., Karpen, I. O., & Hultink, E. J. (2020b). Addressing the design-implementation gap of sustainable business models by prototyping: A tool for planning and executing small-scale pilots. *Journal of Cleaner Production,**255*, 120295.

[CR14] Baldassarre, B., & Micciché, N. (2014). Into the grey: Towards successful implementation and effective evaluation of “Design for Development” projects. *DDR,**2014*, 30.

[CR15] Baldassarre, B., & Saveyn, H. (2023). *A systematic analysis of EU publications on the Circular Economy*. Publications Office of the European Union. 10.2760/36203

[CR16] Barczak, G., Griffin, A., & Kahn, K. B. (2009). Perspective: Trends and drivers of success in NPD practices: Results of the 2003 PDMA best practices study. *Journal of Product Innovation Management,**26*(1), 3–23.

[CR17] Bason, C., and Austin, R. D. (2019). The right way to lead design thinking. *Harvard Business Review, 2019* (March–April), 82–91.

[CR18] Bauman, Z. (2013). *Postmodernity and its discontents*. Wiley.

[CR19] Beverland, M. B., Wilner, S. J., & Micheli, P. (2015). Reconciling the tension between consistency and relevance: Design thinking as a mechanism for brand ambidexterity. *Journal of the Academy of Marketing Science,**43*(5), 589–609.

[CR20] Biedenkopf, K., Van Eynde, S., & Bachus, K. (2019). Environmental, climate and social leadership of small enterprises: Fairphone’s step-by-step approach. *Environmental Politics,**28*(1), 43–63.

[CR21] Blok, V., & Lemmens, P. (2015). The emerging concept of responsible innovation. Three reasons why it is questionable and calls for a radical transformation of the concept of innovation. In B. Koops, I. Oosterlaken, H. Romijn, T. Swierstra, & J. van der Hoven (Eds.), *Responsible innovation 2: Concepts, approaches, and applications* (pp. 19–35). Springer.

[CR22] Bocken, N., Baldassarre, B., Keskin, D., & Diehl, J. C. (2023). Design thinking tools to catalyse sustainable circular innovation. In H. Lehtimäki, L. Aarikka-Stenroos, A. Jokinen, & P. Jokinen (Eds.), *The Routledge handbook of catalysts for a sustainable circular economy* (pp. 1–36). Routledge. 10.13140/RG.2.2.14365.08162

[CR23] British Design Council. (2007). *Eleven lessons: Managing design in eleven global brands. A study of the design process*. British Design Council.

[CR24] British Design Council. (2021). *Beyond net zero*. British Design Council.

[CR25] Brown, T. (2008). Design thinking. *Harvard Business Review,**86*(6), 84–92. 18605031

[CR26] Brown, T. (2009). *Change by design*. HarperCollins e-books.

[CR27] Brown, T., & Martin, R. (2015). Design for action. *Harvard Business Review,**93*(9), 57–64.

[CR28] Brundtland, G. (1987). *Our common future: Report of the 1987 World Commission on Environment and Development*. Retrieved from https://sustainabledevelopment.un.org/content/documents/5987our-common-future.pdf

[CR29] Buchanan, R. (1992). Wicked problems in design thinking. *Design Issues,**8*(2), 5–21.

[CR30] Burget, M., Bardone, E., & Pedaste, M. (2017). Definitions and conceptual dimensions of responsible research and innovation: A literature review. *Science and Engineering Ethics,**23*(1), 1–19. 27090147 10.1007/s11948-016-9782-1

[CR31] Calabretta, G., Durisin, B., & Ogliengo, M. (2011). Uncovering the intellectual structure of research in business ethics: A journey through the history, the classics, and the pillars of Journal of Business Ethics. *Journal of Business Ethics,**104*, 499–524.

[CR32] Calabretta, G., Gemser, G., & Karpen, I. (2016). *Strategic design: Eight essential practices every strategic designer must master*. BIS Publishers.

[CR33] Calabretta, G., Gemser, G., & Wijnberg, N. M. (2017). The interplay between intuition and rationality in strategic decision making: A paradox perspective. *Organization Studies,**38*(3–4), 365–401.

[CR34] Carlgren, L., Rauth, I., & Elmquist, M. (2016). Framing design thinking: The concept in idea and enactment. *Creativity and Innovation Management,**25*(1), 38–57.

[CR35] Carson, R. (1962). *Silent spring*. Fawcett Crest Book.

[CR36] Ceschin, F., & Gaziulusoy, I. (2016). Evolution of design for sustainability: From product design to design for system innovations and transitions. *Design Studies,**47*, 118–163.

[CR37] Chang, Y., Kim, J., & Joo, J. (2013). An exploratory study on the evolution of design thinking: Comparison of Apple and Samsung. *Design Management Journal,**8*(1), 22–34.

[CR38] Ciulli, F., Kolk, A., & Boe-Lillegraven, S. (2020). Circularity brokers: Digital platform organizations and waste recovery in food supply chains. *Journal of Business Ethics,**167*(2), 299–331.

[CR39] Cross, N. (1982). Designerly ways of knowing. *Design Studies,**3*(4), 221–227.

[CR40] Cross, N. (2007). Forty years of design research. *Design Studies,**28*(1), 1–4.

[CR41] Danatzis, I., Karpen, I. O., & Kleinaltenkamp, M. (2022). Actor ecosystem readiness: Understanding the nature and role of human abilities and motivation in a service ecosystem. *Journal of Service Research,**25*(2), 260–280.

[CR42] de Hoop, E., Pols, A., & Romijn, H. (2016). Limits to responsible innovation. *Journal of Responsible Innovation,**3*(2), 110–134.

[CR43] de los Reyes, G., & Scholz, M. (2022). Assessing the legitimacy of corporate political activity: Uber and the Quest for responsible innovation. *Journal of Business Ethics,**184*(1), 51–69. 10.1007/s10551-022-05115-z

[CR44] Dell’Era, C., Magistretti, S., Verganti, R., & Zurlo, F. (2020). Four kinds of design thinking: From ideating to making, engaging, and criticizing. *Creativity and Innovation Management,**29*, 324–344.

[CR45] Dodgson, M., Gann, D. M., & Phillips, N. (Eds.). (2013). *The Oxford handbook of innovation management*. OUP.

[CR46] Dorst, K. (2011). The core of “design thinking” and its application. *Design Studies,**32*(6), 521–532.

[CR47] Du, S. (2021). Reimagining the future of technology: ‘The social dilemma’ review. *Journal of Business Ethics*. 10.1007/s10551-021-04816-1

[CR48] Dunne, D., & Martin, R. (2006). Design thinking and how it will change management education. *Academy of Management Learning and Education,**5*(4), 512–523.

[CR49] Elkington, J. (1998). Partnerships from cannibals with forks: The triple bottom line of 21st century business. *Environmental Quality Management (autumn),**199*, 37–51.

[CR50] Elsbach, K. D., & Stigliani, I. (2018). Design thinking and organizational culture: A review and framework for future research. *Journal of Management,**44*(6), 2274–2306.

[CR51] European Commission. (2013). *Options for strengthening responsible research and innovation*. European Commission. Retrieved from 10.2777/46253

[CR52] European Commission. (2018). *Monitoring the evolution and benefits of responsible research and innovation*. European Commission. Retrieved from https://publications.europa.eu/en/publication-detail/-/publication/2c5a0fb6-c070-11e8-9893-01aa75ed71a1

[CR53] European Commission. (2019a). *The European green deal: Communication from the Commission to the European Parliament, the European Council, the Council, the European Economic and Social Committee and the Committee of the Regions*. European Commission. 10.2307/j.ctvd1c6zh.7

[CR54] European Commission. (2019b). *Research & innovation projects relevant to the circular economy strategy CALLS 2016–2018 HORIZON 2020*. Publications Office of the European Union.

[CR55] European Commission. (2021). *Horizon Europe, the EU research and innovation programme (2021–27)*. Publications Office of the European Union. Retrieved from 10.2777/052084

[CR56] European Parliament. (2018). *Evolution and key data from FP1 to Horizon 2020 in view of FP9 in-depth analysis*. European Parliament. Retrieved from http://www.europarl.europa.eu/thinktank/en/document.html?reference=EPRS_IDA(2017)608697

[CR57] Fassin, Y. (2000). Innovation and ethics ethical considerations in the innovation business. *Journal of Business Ethics,**27*(1), 193–203.

[CR58] Ferraro, F., Etzion, D., & Gehman, J. (2015). Tackling grand challenges pragmatically: Robust action revisited. *Organization Studies,**36*(3), 363–390. 10.1177/0170840614563742

[CR59] Freudenreich, B., Lüdeke-Freund, F., & Schaltegger, S. (2020). A stakeholder theory perspective on business models: Value creation for sustainability. *Journal of Business Ethics,**166*(1), 3–18.

[CR60] Friedman, B., & Hendry, D. G. (2019). *Value sensitive design: Shaping technology with moral imagination*. MIT Press.

[CR61] Fuller, R. B. (1957). *A comprehensive anticipatory design science*. Royal Arch.

[CR62] Fuller, R. B. (1969). *Operating manual for spaceship Earth*. Southern Illinois University Press. 10.2307/812959

[CR63] Gardien, P., Rincker, M., & Deckers, E. (2016). Designing for the knowledge economy: Accelerating breakthrough innovation through co-creation. *Design Journal,**19*(2), 283–299.

[CR64] Garriga, E. (2014). Beyond stakeholder utility function: Stakeholder capability in the value creation process. *Journal of Business Ethics,**120*(4), 489–507.

[CR65] Garriga, E., & Melé, D. (2004). Corporate social responsibility theories: Mapping the territory. *Journal of Business Ethics,**53*(1), 51–71.

[CR66] Gemser, G., & Barczak, G. (2020). Designing the future: Past and future trajectories for design innovation research. *Journal of Product Innovation Management*. 10.1111/jpim.12543

[CR67] Gemser, G., & Perks, H. (2015). Co-creation with customers: An evolving innovation research field. *Journal of Product Innovation Management,**32*(5), 660–665.

[CR68] George, G., Howard-Grenville, J., Joshi, A., & Tihanyi, L. (2016). Understanding and tackling social grand challenges through management research. *Academy of Management Journal,**59*(6), 1880–1895.

[CR69] George, G., Schillebeeckx, S., & Liak, T. L. (2015). The management of natural resources: An overview and research agenda. *Academy of Management Journal,**58*(6), 1595–1613.

[CR70] Godelnik, R. (2017). Millennials and the sharing economy: Lessons from a ‘buy nothing new, share everything month’ project. *Environmental Innovation and Social Transitions,**23*, 40–52.

[CR71] Goffin, K., Åhlström, P., Bianchi, M., & Richtnér, A. (2019). Perspective: State-of-the-art: The quality of case study research in innovation management. *Journal of Product Innovation Management,**36*(5), 586–615.

[CR72] Greenwood, M., & Freeman, R. E. (2018). Deepening ethical analysis in business ethics. *Journal of Business Ethics,**147*(1), 1–4.

[CR73] Gruber, M., De Leon, N., George, G., & Thompson, P. (2015). Managing by design: From the editors. *Academy of Management Journal,**58*(1), 1–7.

[CR74] Gutierrez, L., Montiel, I., Surroca, J. A., & Tribo, J. A. (2022). Rainbow wash or rainbow revolution? Dynamic stakeholder engagement for SDG-driven responsible innovation. *Journal of Business Ethics,**180*(4), 1113–1136. 10.1007/s10551-022-05190-2 10.1007/s10551-022-05190-2 PMC9297266 35873084

[CR75] Hahn, T., Pinkse, J., Preuss, L., & Figge, F. (2015). Tensions in corporate sustainability: Towards an integrative framework. *Journal of Business Ethics,**127*, 297–316.

[CR76] Hamington, M. (2019). Integrating care ethics and design thinking. *Journal of Business Ethics,**155*(1), 91–103.

[CR77] Hardin, G. (1968). The tragedy of the commons. *Science,**162*(3859), 243–1248. 5699198

[CR78] Hartley, K., Baldassarre, B., & Kirchherr, J. (2023). Circular economy as crisis response. *Journal of Cleaner Production,**434*, 140140.

[CR79] Hey, J. H., Joyce, C. K., & Beckman, S. L. (2007). Framing innovation: Negotiating shared frames during early design phases. *Journal of Design Research,**6*(1–2), 79–99.

[CR80] Hillgren, P. A., Seravalli, A., & Emilson, A. (2011). Prototyping and infrastructuring in design for social innovation. *CoDesign,**7*(3–4), 169–183.

[CR81] Johansson-Sköldberg, U., Woodilla, J., & Çetinkaya, M. (2013). Design thinking: Past, present and possible futures. *Creativity and Innovation Management,**22*(2), 121–146.

[CR82] Johnston, A., Amaeshi, K., Adegbite, E., & Osuji, O. (2021). Corporate social responsibility as obligated internalisation of social costs. *Journal of Business Ethics,**170*, 39–52.

[CR83] Karpen, I. O., Gemser, G., & Calabretta, G. (2017). A multilevel consideration of service design conditions. *Journal of Service Theory and Practice,**27*(2), 384–407.

[CR84] Klaassen, P., Kupper, F., Vermeulen, S., Rijnen, M., Popa, E., & Broerse, J. (2017). The conceptualization of RRI: An iterative approach. In *Responsible innovation 3: A European agenda?* (pp. 69–92). Springer.

[CR85] Klenner, N. F., Gemser, G., & Karpen, I. O. (2022). Entrepreneurial ways of designing and designerly ways of entrepreneuring: Exploring the relationship between design thinking and effectuation theory. *Journal of Product Innovation Management,**39*(1), 66–94.

[CR86] Knapp, J., Zeratsky, J., & Kowitz, B. (2016). *Sprint: How to solve big problems and test new ideas in just five days*. Simon and Schuster.

[CR87] Kolko, J. (2015). Design thinking comes of age. *Harvard Business Review,**93*(9), 66–71.

[CR88] Kumar, V., & Holloway, M. (2009). How tangible is your strategy? How design thinking can turn your strategy into reality. *Journal of Business Strategy,**30*(2/3), 50–56.

[CR89] Liedtka, J. (2015). Perspective: Linking design thinking with innovation outcomes through cognitive bias reduction. *Journal of Product Innovation Management,**32*(6), 925–938.

[CR90] Liedtka, J., King, A., & Bennett, K. (2013). *Solving problems with design thinking: Ten stories of what works*. Columbia University Press.

[CR91] Liedtka, J., Salzman, R., & Azer, D. (2017). Design thinking for the greater good: Innovation in the social sector. In *Foundation review* (Vol. 10, Issue 1). Columbia University Press. 10.9707/1944-5660.1411

[CR92] Linton, J. D., & Thongpapanl, N. (2004). Perspective: Ranking the technology innovation management journals. *Journal of Product Innovation Management,**21*(2), 123–139.

[CR93] Lubberink, R., Blok, V., van Ophem, J., & Omta, O. (2017). Lessons for responsible innovation in the business context: A systematic literature review of responsible, social and sustainable innovation practices. *Sustainability (switzerland),**9*(5), 721.

[CR94] MacInnis, D. J. (2011). A framework for conceptual contributions in marketing. *Journal of Marketing,**75*(4), 136–154.

[CR95] Magistretti, S., Ardito, L., & Petruzzelli, A. M. (2021). Framing the microfoundations of design thinking as a dynamic capability for innovation: Reconciling theory and practice. *Journal of Product Innovation Management,**38*(6), 645–667.

[CR96] Manzini, E. (2017). Designing coalitions: Design for social forms in a fluid world. *Strategic Design Research Journal,**10*(2), 187–193.

[CR97] Martin, R. (2009). *The design of business: Why design thinking is the Next Competitive Advantage*. Harvard Business School Press.

[CR98] Martin, R. (2010). Design thinking: Achieving insights via the “knowledge funnel. *Strategy and Leadership,**38*(2), 37–41.

[CR99] Matthews, L., Power, D., Touboulic, A., & Marques, L. (2016). Building bridges: Toward alternative theory of sustainable supply chain management. *Journal of Supply Chain Management,**52*(1), 82–94.

[CR100] Mazzucato, M. (2018). Mission-oriented innovation policies: Challenges and opportunities. *Industrial and Corporate Change,**27*(5), 803–815.

[CR101] McGann, M., Blomkamp, E., & Lewis, J. M. (2018). The rise of public sector innovation labs: Experiments in design thinking for policy. *Policy Sciences,**51*(3), 249–267.

[CR102] Meadows, D. H., Meadows, D. L., Randers, J., & Behrens, W. W. (1972). *The limits to growth: A report to The Club of Rome*. Universe Books.

[CR103] Micheli, P., Perks, H., & Beverland, M. B. (2018). Elevating design in the organization. *Journal of Product Innovation Management,**35*(4), 629–651.

[CR104] Micheli, P., Wilner, S. J. S., Bhatti, S. H., Mura, M., & Beverland, M. B. (2019). Doing design thinking: Conceptual review, synthesis, and research agenda. *Journal of Product Innovation Management,**36*(2), 124–148.

[CR105] Mink, A., Parmar, V. S., & Kandachar, P. V. (2014). Responsible design and product innovation from a capability perspective. In *Responsible innovation 1* (pp. 113–148). Springer.

[CR106] Mintrom, M., & Luetjens, J. (2016). Design thinking in policymaking processes: Opportunities and challenges. *Australian Journal of Public Administration,**75*(3), 391–402.

[CR107] Müller-Seitz, G., & Weiss, W. (2021). Design thinking as an Agile Panacea? Towards a symbiotic understanding of design thinking and organizational culture. In *The Agile imperative: Teams, organizations and society under reconstruction?* (pp. 115–137). Macmillan.

[CR108] Nyffenegger, R., Baldassarre, B., & Bocken, N. (2023). Circular business models and supporting policies for reusing of photovoltaic modules in the EU. In K. Niinimäki & K. Cura (Eds.), *Product lifetimes and the environment 2023* (pp. 738–744). Aalto University Publication Series. 10.13140/RG.2.2.24431.41124

[CR109] Okimoto, T. G. (2014). Toward more interesting research questions: Problematizing theory in social justice. *Social Justice Research,**27*, 395–411.

[CR110] Owen, R., Pansera, M., Macnaghten, P., & Randles, S. (2021). Organisational institutionalisation of responsible innovation. *Research Policy,**50*(1), 104132.

[CR111] Pandza, K., & Ellwood, P. (2013). Strategic and ethical foundations for responsible innovation. *Research Policy,**42*(5), 1112–1125.

[CR112] Papanek, V. (1971). *Design for the real world: Human ecology and social change*. Thames and Hudson.

[CR113] Paton, B., & Dorst, K. (2011). Briefing and reframing: A situated practice. *Design Studies,**32*(6), 573–587.

[CR114] Plattner, H., Meinel, C., & Leifer, L. (2009). Design thinking research. In H. Plattner, C. Meinel, & L. Leifer (Eds.), *Design thinking: Understand–improve–apply. *Springer.

[CR115] Porter, M., & Kramer, M. R. (2011). Creating shared value. *Harvard Business Review,**89*(1–2), 62–77. 10662001

[CR116] Prahalad, C. K. (2012). Bottom of the pyramid as a source of breakthrough innovations. *Journal of Product Innovation Management,**29*(1), 6–12.

[CR117] Prestes Joly, M., Teixeira, J. G., Patrício, L., & Sangiorgi, D. (2019). Leveraging service design as a multidisciplinary approach to service innovation. *Journal of Service Management,**30*(6), 681–715.

[CR118] Quint, E., Gemser, G., & Calabretta, G. (2022). *Design leadership ignited: Elevating design at scale*. Stanford University Press.

[CR119] Reckwitz, A. (2002). Toward a theory of social practices: A development in culturalist theorizing. *European Journal of Social Theory,**5*(2), 243–263.

[CR120] Reinecke, J., & Ansari, S. (2016). Taming wicked problems: The role of framing in the construction of corporate social responsibility. *Journal of Management Studies,**53*(3), 299–329.

[CR121] Rindova, V. P., & Martins, L. L. (2021). Futurescapes: Imagination and temporal reorganization in the design of strategic narratives. *Strategic Organization*. 10.1177/1476127021989787

[CR122] Rittel, H., & Webber, M. (1973). Dilemmas in a general theory of planning. *Policy Sciences,**4*(2), 155–169.

[CR123] Robaey, Z., & Simons, A. (2015). Responsible management of social experiments: Challenges for policymaking. In *Responsible innovation 2: Concepts, approaches, and applications* (pp. 87–103). Springer.

[CR124] Rockström, J., Steffen, W., Noone, K., Asa Persson, F., Stuart Chapin, I. I. I., Lambin, E. F., Lenton, T. M., Scheffer, M., Folke, C., Schellnhuber, H. J., Nykvist, B., de Wit, C. A., Hughes, T., van der Leeuw, S., Rodhe, H., Sörlin, S., Snyder, P. K., Costanza, R., Svedin, U., … Foley, J. A. (2009). A safe operating space for humanity. *Nature,**461*(7263), 472–475. 10.1038/461472a 19779433

[CR125] Roos, J., Victor, B., & Statler, M. (2004). Playing seriously with strategy. *Long Range Planning,**37*(6), 549–568.

[CR126] Sanders, E., & Stappers, P. J. (2008). Co-creation and the new landscapes of design. *CoDesign,**4*(1), 5–18.

[CR127] Scherer, A. G., & Palazzo, G. (2011). The new political role of business in a globalized world: A review of a new perspective on CSR and its implications for the firm, governance, and democracy. *Journal of Management Studies,**48*(4), 899–931.

[CR128] Scherer, A. G., Rasche, A., Palazzo, G., & Spicer, A. (2016). Managing for political corporate social responsibility: New challenges and directions for PCSR 2.0. *Journal of Management Studies,**53*(3), 273–298.

[CR129] Schön, D. A. (1983). *The reflective practitioner: How professionals think in action*. Basic Books.

[CR130] Schön, D. A. (1988). Designing: Rules, types and worlds. *Design Studies,**9*(3), 181–190.

[CR131] Schön, D. A. (1992). Design as reflective conversation with the materials of a design situation. *Knowledge-Based Systems,**5*(1), 3–14.

[CR132] Schön, D. A., & Rein, M. (1994). *Frame reflection: Toward the resolution of intractable policy controversies*. Basic Books.

[CR133] Simon, H. A. (1968). *The sciences of the artificial*. MIT.

[CR134] Simon, H. A. (1995). Problem forming, problem finding and problem solving in design. In *Design & systems* (pp. 245–257). Transaction Publishers.

[CR135] Sparks, J. R., & Pan, Y. (2010). Ethical judgments in business ethics research: Definition, and research agenda. *Journal of Business Ethics,**91*, 405–418.

[CR136] Staton, B., Kramer, J., Gordon, P., & Valdez, L. (2016). From the technical to the political: Democratizing design thinking. Paper presented at Contested Cities to Global Urban Justice conference, July, Madrid.

[CR137] Steen, M., Sand, M., & Van de Poel, I. (2021). Virtue ethics for responsible innovation. *Business and Professional Ethics Journal,**40*(2), 243–268.

[CR138] Stilgoe, J., Owen, R., & Macnaghten, P. (2013). Developing a framework for responsible innovation. *Research Policy,**42*(9), 1568–1580.

[CR139] Thongpapanl, N. T. (2012). The changing landscape of technology and innovation management: An updated ranking of journals in the field. *Technovation,**32*(5), 257–271.

[CR140] United Nations. (2015). *Transforming our world: The 2030 agenda for sustainable development 2015*. United Nations.

[CR141] Verganti, R. (2008). Design, meanings and radical innovation: A meta-model and a research agenda. *Journal of Product Innovation Management,**25*(5), 436–456.

[CR142] Verganti, R. (2009). *Design driven innovation: Changing the rules of competition by radically innovating what things mean*. Harvard Business Press.

[CR143] Verganti, R. (2017). *Overcrowded: Designing meaningful products in a world awash with ideas*. *Overcrowded: Designing meaningful products in a world awash with ideas*. MIT.

[CR144] Verganti, R., Dell’Era, C., & Swan, K. S. (2021). Design thinking: Critical analysis and future evolution. *Journal of Product Innovation Management,**38*(6), 603–622.

[CR145] Vink, J., & Koskela-Huotari, K. (2022). Building reflexivity using service design methods. *Journal of Service Research,**25*(3), 371–389.

[CR146] Viswanathan, M., & Sridharan, S. (2012). Product development for the BoP: Insights on concept and prototype development from university-based student projects in India. *Journal of Product Innovation Management,**29*(1), 52–69.

[CR147] Vliegenthart, R., & Roggeband, C. (2007). Framing immigration and integration: Relationships between press and parliament in the Netherlands. *International Communication Gazette,**69*(3), 295–319.

[CR148] Voegtlin, C., & Scherer, A. G. (2017). Responsible innovation and the innovation of responsibility: Governing sustainable development in a globalized world. *Journal of Business Ethics,**143*(2), 227–243.

[CR149] Von Schomberg, R. (2011). Prospects for technology assessment in a framework of responsible research and innovation. In M. Dusseldorp & R. Beecroft (Eds.), *Technikfolgen abschätzen lehren: Bildungspotenziale transdisziplinärer Methoden*. Vs Verlag.

[CR150] Von Schomberg, R. (2013). A vision of responsible research and innovation. In R. Owen, J. Bessant, & M. Heintz (Eds.), *Responsible innovation. *Wiley.

[CR151] Waheed, A., & Zhang, Q. (2022). Effect of CSR and ethical practices on sustainable competitive performance: A case of emerging markets from stakeholder theory perspective. *Journal of Business Ethics,**175*(4), 837–855.

[CR152] Werhane, P. H., & Freeman, R. E. (1999). Business ethics: The state of the art. *International Journal of Management Reviews,**1*(1), 1–16.

[CR153] Windahl, C., Karpen, I. O., & Wright, M. R. (2020). Strategic design: Orchestrating and leveraging market-shaping capabilities. *Journal of Business and Industrial Marketing,**35*(9), 1413–1424.

[CR154] Wohlin, C. (2014). Guidelines for snowballing in systematic literature studies and a replication in software engineering. In *Proceedings of the 18th international conference on evaluation and assessment in software engineering—EASE’14* (pp. 1–10). Retrieved from http://dl.acm.org/citation.cfm?doid=2601248.2601268

[CR155] Zahra, S. A., Gedajlovic, E., Neubaum, D. O., & Shulman, J. M. (2009). A typology of social entrepreneurs: Motives, search processes and ethical challenges. *Journal of Business Venturing,**24*(5), 519–532.

