# Supplementary material for: Responsible Design Thinking for Sustainable Development: Critical Literature Review, New Conceptual Framework, and Research Agenda
Source: J Bus Ethics. 2024 Feb 5;195(1):25–46. doi: 10.1007/s10551-023-05600-z (PMC11534984; doi:10.1007/s10551-023-05600-z)
Supplement: Supplementary file 1 — Supplementary file1 (DOCX 74 KB) [file 10551_2023_5600_MOESM1_ESM.docx]

**APPENDIX A**

**Analysis and categorization of selected design thinking articles**

|  |  | **Design Thinking** | | | |
| --- | --- | --- | --- | --- | --- |
| **Journal** | **Author(s)** | **Main focus** | **Economic impact** | **Social impact** | **Environmental impact** |
| European Management Review | Agafonow (2019) | Economic, social | Makes the case that DT can support from a methodological perspective social enterprise research in producing knowledge (p.799) about pursuing profit effectively (p.803) | Makes the case that DT can support from a methodological perspective social enterprise research in producing knowledge (p.799) about fostering social gains (p.803) | - |
| Academy of Management Perspectives | Aguinis et al. (2020) | Social | Mentions that organizations may benefit by the knowledge produced through DT in business schools (p.171) | Makes the case that infusing a DT perspective into management research can make the latter more effective in investigating a desirable future for society (p.170-171) | - |
| California Management Review | Appleyard et al. (2020) | Economic | Examine how a business organization incorporates DT principles in R&D as a way to regain market leadership (p.12), for example through creative forbearance in profit planning (p.22). | - | - |
| Technovation | Bartoloni et al., 2022 | Social |  | Develop and apply a framework where DT facilitates the integration of social goals in technological innovation by regulating the interaction among subsystems, ensuring stakeholders’ collective participation, and feeding the circuit of knowledge creation and sharing (p.9-10). |  |
| Harvard Business Review | Bason and Austin (2019) | Social | - | Use several examples of applying DT in the public sector as a way to improve public services (e.g., health insurance, food charity) and make a positive social impact (p.82-91) | - |
| California Management Review | Beckman  (2020) | Economic | Introduces and discusses DT as a problem-solving activity occurring primarily in an economic context (p.144), useful for example to try out new business models (p.157) | Briefly mentions that observing is part of DT, and that this entails paying attention to social trends (p.150) and that DT should be applied also by the government in the public sector (p.158) | Briefly mentions that observing is part of DT, and that this entails paying attention to environmental trends (p.150) |
| California management review | Beckman and Barry (2007) | Economic | Introduce and discuss DT primarily as an approach to lead in innovation and gain competitive advantage through organizational learning (p.25) | Provide a little background on DT, briefly acknowledging that in the past this approach was conceptualized as a way to ensure social equity and pluralism (p.26). | In a few lines, briefly mentions student projects applying DT to consider product life cycle and environmental sustainability (p.41) |
| International Journal of Operations & Production Management | Beltagui (2018) | Economic | Make the case that DT capabilities can help firms to develop service offering and gain competitive advantage through business model innovation (p.1040), which allows to improve value creation, revenue capture and profit generation (p.1042). Discusses sustainability exclusively from a financial perspective (p.1052) | - | - |
| Journal of the Academy of Marketing Science | Beverland et al. (2015) | Economic | Present DT as a mechanism to promote and enable the integration of brand consistency and relevance, which is functional for brand ambidexterity and ultimately to sustain organizational growth and brand equity (p. 589). | Presents human-centeredness as an attribute of DT (p.593) | Presents a few company cases where DT was able to uncover the tension between financial performance and environmental sustainability (p.596) |
| Academy of Management Learning & Education | Boni et al. (2009) | Economic, social | Present DT as an essential perspective for the commercialization of innovation to be incorporated in the entrepreneurship curricula of business schools (p.407). | Present a few cases of business school student projects where DT and entrepreneurship takes place as way to make a positive social impact (e.g. eradication of malaria) next to pursuing profit (p.412-413) | - |
| Harvard Business Review | Brown (2008) | Economic, social | Presents DT to business leaders as a source of competitive advantage (p.2), and explain that historically this has been the case since the 1950s (p.3) | Uses the case of an eye care provider targeting poor people in India to illustrate that DT can make a social impact (p.6-7) | - |
| Journal of Product Innovation Management | Brown and Katz (2011) | Social, economic,  environmental | Explain that DT is a set of skills that can be applied to solve issues of organizational management and strategic innovation (p.381) to outrun competitors (p.383) | Explain that DT is a set of skills that are relevant especially to solve complex social problems (p.381), making a link to the United Nations Millennium goals such as eradicating poverty and promoting gender equality (p.383) | Mentions that some of the great challenges lying ahead are environmental, such as the melting glaciers of Greenland, implying that they may be addressed through DT (p.383) |
| Harvard Business Review | Brown and Martin (2015) | Social, economic | Explain that DT is behind the commercial success of many products and services (p.57) because it allows to develop better business propositions (p.67) | Make the case that the scope of DT is addressing wicked problems (p.58), providing the example of a large bank innovating experimentally to foster economic development while solving social challenges in Peru (p.64-71) |  |
| California Management Review | Björklund et al. (2020) | Economic, social, environmental | Introduce DT as a source of competitive advantage for companies and explore through interviews with designers how design capabilities can be effectively implemented in business organizations (p.100) to increase profitability and brand value (p.101) | Conclude by explaining that organizations are responsible for the outcome of their innovation process (p.119), that DT driven organizations are more likely to innovate responsibly and that socially sustainable innovation is the next key question for organizations (p.120) | Conclude stating that environmentally sustainable innovation is the next key question for organizations (p.120) |
| Technology Analysis & Strategic Management | Cagnin (2018) | Social, environmental, economic | Discuss the application of DT in combination with the “discipline of anticipation” in the context of a research organization working for the Brazilian government aiming to identify key questions and themes to make future cities stronger from an economic standpoint (p.524-539) | Discuss the application of DT in combination with the “discipline of anticipation” in the context of a research organization working for the Brazilian government aiming to identify key questions and themes to make future cities more socially sustainable (p.524-539), such as access to services for low-income people (p.529) | Discuss the application of DT in combination with the “discipline of anticipation” in the context of a research organization working for the Brazilian government aiming to identify key questions and themes to make future cities more environmentally sustainable (p.524-539), such as the use of renewable energy (p.529) |
| Industrial Marketing Management | Cankurtaran and Beverland (2020) | Social, economic,  environmental | Explain that DT is an essential approach for organizations not only to enhance competitiveness (p.258) but also to become more resilient and survive when markets are disrupted by external calamitous forces (p.255) | Explicitly explains that DT is suitable to address wicked problems in times of crisis (p.255-260), providing relevant examples of DT driven social innovation performed by private and public organizations (p. 257) | Mention that future crises will be triggered by environmental issues such as climate change, making DT an increasingly relevant problem-solving approach in this regard (p.256) |
| Creativity and Innovation Management | Carlgren et al. (2016) | Economic | Present DT as driver for competitive advantage and discuss problems related to its implementation in business organizations (p.344) | - | - |
| Journal of Product Innovation Management | Carlgren et al., 2021 | Economic | Take a culture perspective on DT and identify the its cultural characteristics of DT and the related challenges. Argue that overcoming the challenges deriving from misfit between DT cultural characteristics and organizational culture is essential for implementing DT and ultimately creating value (p. 58) | - | - |
| Design Management Review | Carr et al. (2010) | Economic | Investigate evidence around the tangible impacts of implementing DT in large business corporations (p.59-63) and pose future questions concerning economic performance (p.63) | - | - |
| Design Management Journal | Chang et al. (2013) | Economic | Focus on understanding and applying DT in business organization by comparing Apple and Samsung (p.22), explaining that this is ultimately relevant to outperform competitors in innovation, increasing sales and revenue (p.23) | - | - |
| Journal of Marketing Management | Chen and Venkatesh (2013) | Economic | Examine how DT is implemented into business organizations as a way to gain competitive advantage through a unified brand experience and differentiation from competitors, achieved for example through improved product development, packaging and brand logo (p.1680) | Briefly mention that improve human and social conditions is in principle within the scope of DT (p.1682) | - |
| Design Management Review | Clark and Smith (2008) | Economic | Use the case of IBM (p.11) to argue that DT is one the most important drivers to gain competitive advantage in innovation by boosting managerial emotional, integral and experiential intelligence (p.9), which in turn drive profit generation (p.10) | - | - |
| Creativity and Innovation Management | Coco et al. (2020) | Economic, social | Present DT as a strategic resource for competitive success (p.1) and discuss how it can be successfully infused into the culture of an organization facing resource constraints and competing demands (p.10-13) | Use student project performed within an innovation management course to illustrate that the outcomes of applying DT have social relevance (p.9-10) | - |
| Design Management Review | Collins (2013) | Economic, social | Presents DT as a source competitive advantage for business (p.36) and then explains what this approach is about (p.37) and why it is not implemented (p.39) | Proposes some ideas about what DT may be in the future (p.37), mentioning the importance of using it to address social challenges in developing countries, such as access to clean water and a safe cooking environment (p.38) | - |
| Design Management Review | Cooper et al. (2009) | Economic, social | Introduce DT as a tool traditionally used to help business organizations improving product development and marketing (p.48), and argue that nowadays using DT in the context of strategy is essential to achieve competitive advantage (p.46) | Briefly mentions that the frontier of applying DT should be transforming the entire organization and, beyond that, society (p.54) | Briefly mentions that DT is relevant to address also environmental issue, next to economic, social and political ones (p.54) |
| Design Management Review | Dalton and Kahute (2017) | Economic | Make the case that business organizations should leverage DT through empathy as a way to achieve customer closeness (p.20), which ultimately results in customer advocacy, better value creation, minimization of economic risk, competitive advantage and growth (p.26-27) | - | - |
| Creativity and Innovation Management | Dell'Era et al. (2020) | Economic | Introduce DT as a matter of tangible interests in the competitive arena management and business world (p.324, 336) and analyze 47 consultancy projects as a way to interpret DT in four different ways: creative problem solving, sprint execution, creative confidence, and innovation of meaning (p.324) | Mention that a human approach is the backbone of DT (p.326) | - |
| California Management Review | Dong et al. (2016) | Economic | Discuss DT in relation to generative sensing, a particular type of dynamic capability that helps companies in generating strategic options to outperform competitors (p.97) | - | - |
| Design Management Review | Drews (2009) | Economic | Uses expert interviews to identify ways in which DT as a management practice can benefit business. Benefits include ability to stay relevant in changing environments (p.41) and ability to better understand customers and thus come up with better offerings (p.42) | Briefly mention that DT can eventually be used to re-shape the economic system and society (p.44) | Briefly mention that DT can eventually be used to address sustainability issues (p.44) |
| Academy of Management Learning & Education | Dunne and Martin (2006) | Economic, social | Open the article presenting DT as a critical component for business competitiveness, that multinational companies should leverage to become leaders through the creation of better products and services (p.512) | DT integrative/system thinking automatically leads to consider the needs of customers stakeholders, shareholders and society at large, thus bringing ethical and social values into their solutions (p.520). Managers of the future (with DT education in their MBAs) will consider the consequences of their choices on the systems as a whole - including on issues like poverty, the environment and geopolitics (p.520) | Managers of the future (with DT education in their MBAs) will consider the consequences of their choices on the systems as a whole - including on issues like poverty, the environment and geopolitics (p.520) |
| Journal of Management | Elsbach and Stigliani (2018) | Economic | Through a literature review, establish a link between DT tools and organizational culture and suggest that this connection is critical for companies to reach competitive advantage (p.25) | - | Mention that in the past years there has been an excessive emphasis on the human-centered focus of DT, which lead to disregard other strategic issues like the focus on environmental sustainability (p.27) |
| Research Technology Management | Euchner (2012) | Economic | Discusses how the intuitive insights of DT complement managers’ analytical way of thinking to create breakthrough solutions and, thus, lead to competitive advantage (p.10) | - | - |
| Design Management Review | Fixson and Rao (2014) | Economic | Indicate that DT principles like experimentation and iteration help managers to better deal with uncertainty and, thus, formulate emerging strategies to better perform in the market (p.48) | - | - |
| Journal of Business Strategy | Fraser (2007) | Economic | Argues that, when companies use DT methods and mindset for shaping strategy and business model, this can lead to innovative growth strategy and, ultimately, to competitive advantage that is sustainable from a financial standpoint (p.67) | - | - |
| Design Management Review | Fraser (2009) | Economic, social | Positions DT as a core competence for firms to create new business model (p.57) and gain competitive advantage | Explicitly mentions than new business models have to be developed through DT as a response to higher expectations of corporate social responsibility (p.57) | - |
| Design Management Review | Gardien and Gilsing (2013) | Economic, social | Argues that DT must become a core function of organizations as a way to improve business performance in multiple areas such as brand management, engineering and talent scouting (p.58) | Argues that DT is as well a vehicle for social and cultural change (p.66), using positive the example of healing environments as a way to illustrate this (p.63) | - |
| Qualitative Market Research | Garrett and Wrigley (2019) | Economic, environmental | Explore how a DT process incorporating both deep customer insights and traditional market research can help companies finding new opportunities and developing new business models (p.457) in a competitive market environment (p.464) | - | Link new business models to innovative collaborative consumption patterns in the mobility sector (p.463), which has a positive impact from an environmental perspective |
| Academy of Management Learning & Education | Glen et al. (2014) | Economic | Argues that DT is a source of business competitiveness (p.660), therefore it should be incorporated in the curriculum of business schools (p.653) | - | - |
| International Journal of Management Education | Glen et al. (2015) | Economic, social | Present DT as an approach to innovation that uses deep understanding of people and iteration to support managers and entrepreneurs in creating new sources of value in uncertain business contexts (p.183) | Elaborates on why teaching DT in business schools is important for the personal and professional development of the students (p.190) | - |
| Research Technology Management | Gobble (2014) | Economic | Connects DT to the ability of firms to stay competitive in the digital era (p.59-61) | - | - |
| Academy of Management Journal | Gruber et al. (2015) | Economic, social | Present DT as a source of competitive advantage for business (p.1) providing examples on how this is relevant in the new, digital, experience economy (p.5) | Present DT as a successful approach for the government to improve public services (p.1), also discussing work-related social values and individual well-being (p.6-7) | - |
| MIT Sloan Management Review | Guterman (2009) | Economic | Interviews two business leaders on what is the business value of DT (p.39), and one of the two explicitly relates DT to competitive advantage (p.42) | - | - |
| Journal of Business Ethics | Hamington  (2019) | Social | - | Discuss DT in combination with care ethics to propose the concept of” caring design” (p.91), which holds value for solving the systemic problems of society (p.98) | - |
| Technovation | Hobday et al. (2012) | Economic,  social | Discuss the role of DT in policy making as a way to promote national economic growth (p.272) | Explicitly connects DT to wicked policy making problems and to the improvement of society (p. 280) providing the example of creating sustainable cities in developing countries to improve the livelihood of people (p.278) | - |
| Harvard Business Review | Ignatius (2015) | Economic | Examines the role of “chief design officer” in business organizations | - | - |
| Journal of Business Strategy | Jayakumar et al. (2019) | Social, environmental | - | Present and discuss DT as a winning strategy that the nonprofit sector can use to solve complex social challenges (p.28) | Discuss the case of an NGO, which has used DT to address the issue of water scarcity in the Thar Desert (p.28) |
| Creativity and Innovation Management | Johansson-  Skoldberg et al. (2013) | Economic, social | Review the management discourse on DT and its relevance to address organizational problems in business (p.128) | Review the design discourse on DT, and its relevance to address wicked problems, specifying that they are social systems problems (p.125) | - |
| Journal of Business Strategy | Junginger (2007) | Social | - | Argues around the importance to design with” heart, hand and mind” in order to develop products that are good for people (p.59) | - |
| Journal of Product Innovation Management | Klenner et al., (2021) | Economic | Build a strong theoretical foundation for the effectiveness of DT by integrating abstract effectual theorizing with concrete designerly ways of working. The identified DT practices improve entrepreneurial innovation and new venture creation (p.87) | - | - |
| California Management Review | Knight et al. (2020) | Economic | Makes the case that integrating DT in business strategy is crucial to achieve competitive advantage (p.31) | - | - |
| Harvard Business Review | Kolko (2015) | Economic | Argue that DT must be a core competence for companies to compete in innovation (p.5) and ultimately to survive (p. 4) | Mentions that DT can improve the way people interact with technologies (p.4) | - |
| Design Management Journal | Koomans and Hilders (2016) | Economic, social | Argue that DT can be leveraged to create new business models and competing value propositions (p.48) | Discuss DT in the context of healthcare as a way to improve the sector (p.43-54) and create patient-oriented innovations (p. 45) | - |
| Journal of Business Strategy | Kumar and Holloway (2009) | Economic | Discuss DT as an approach to make business strategy tangible (p.50), concretize emerging business models and identify their competitive differentiators (p.53) | Briefly mentions that DT allows to understand social factors (p.51) | - |
| Creativity and Innovation Management | Kummitha (2019) | Social, economic | Explains that DT has been mostly leveraged as a strategy to advance product and service creation and delivery in for‐profit organizations,while there has been little focus on DT in social organizations (p.103) | Conducted a qualitative study in four social organizations in India to better understand the role of DT in this context (p.105-110) as a way to reduce the gap between designers and communities (p. 101) and foster organizational sustainability (p.110) | - |
| Social Enterprise Journal | Kummitha (2018) | Social, economic | Explains that DT h apas been studied as an approach to boost organizational performance over competitors (p.94) | Focuses on DT in the context of social entrepreneurship to understand how it can be leveraged to address social challenges (p.92-104) | Briefly mentions that DT is relevant to address environmental challenges (p.95) |
| Academy of Management Learning & Education | Kurtmollaiev et al. (2018) | Economic | Explain that DT can be used to train employees with the goal of improving team innovation output, and team operational capability (p. 184, resulting in a more competitive business (p.190) | Briefly mentions that DT has been also applied to social policies (p.186) |  |
| Strategic Change | Lages et al. (2020) | Economic, social | Investigate the role of DT in the context of partnerships, as a way to strategically balance cooperation and competition (p.195) | Argues in the conclusion that sustainability must be considered in combination with DT (p.210) to create shared value for society (p.195) | - |
| Strategy & Leadership | Leavy (2010) | Economic | Argues that DT’s creative principles long associated with the design function can be applied to business management and strategy development (p. 5-6), using the case of McDonalds to illustrated how the company gained first mover advantage over competitors (p.8) | - | - |
| Strategy & Leadership | Leavy (2012) | Economic | Explains that DT is relevant to gain competitive advantage through co-creation with customers (p.25) and quotes Brown saying that DT must be used by business leaders (p.26) | Makes a brief reference to Brown and IDEO using DT to improve the healthcare sector (p.26) | Makes a brief reference to Brown arguing that DT can be leveraged to address climate change (p.26) |
| Journal of Business Strategy | Lewis et al. (2017) | Economic | Explain how DT can be used to create successful interorganizational relationships (p.20), which are essential to compete on price and quality on the market (p.27) | - | - |
| Strategy & Leadership | Liedtka (2014) | Economic,  social | Introduces DT as a source of competitive advantage for business (p.40) and explores what it is how it works to discover and exploit business opportunities (p.43) | Explains that DT can be used also is the social and governmental sector (p.41), mentioning one example of an entrepreneur using it to improve healthcare (p.43) | - |
| Journal of Product Innovation Management | Liedtka (2015) | Economic | Explains that DT can foster innovation and growth in business organizations by helping managers to reduce their cognitive biases (p.925) | Briefly mentions that using DT to reduce cognitive biases can result in prosocial motivation to solve the problems of other people (p.933) | - |
| California Management Review | Liedtka (2020) | Economic,  social | Makes the case that DT is a “social technology” that allows to overcome psychological barriers in the innovation process, helping companies to acquire dynamic capabilities, which are essential to sense and seize new business opportunities (p.53) | Discuss multiple cases where DT in applied to make a positive social impact by businesses, the public sector as well as nonprofit organizations (p.60-62) | - |
| Strategy & Leadership | Liedtka and Kaplan (2019) | Economic | DT can improve organizational strategy by helping to see opportunities, fostering learning in action, supporting portfolio management, facilitating change and transforming the business model (p.3) | Mention in a footnote that DT can be applied in the social sector to achieve a “greater good” (p.10) | - |
| Design Management Review | Liedtka and Ogilvie (2012) | Economic | Makes the case that managers can use DT tools to create more value for customers (p.7-8) | - | - |
| Public Performance & Management Review | Liedtka et al. (2020) | Social | Explains that DT can lead to improved customer satisfaction, employee engagement, and cost avoidance for organizations (p.173) | Discuss how DT can be applied by the US government as human-centered approach to improve lives (p.157-167) | - |
| Design management review | Lockwood (2010) | Economic | Interviews a designer enquiring how the impact of DT as a source of competitive advantage in business can me measured (p.17-24) | Briefly mentions the example of a furniture system developed by Herman Miller healthcare for doing good in society while pursuing an opportunity for the company (p.18) | - |
| Industrial Marketing Management | Luotola et al. (2017) | Economic | Explain that DT and actor-network theory to explore how certainty evolves between a seller and the buyer (p.59) ,while arguing that DT, if managed properly, can result in competitive advantage (p.62) | - | - |
| Creativity and Innovation Management | Magistretti et al. (2022) | Economic | Analyze managers’ perceptions of DT as an approach to achieve business innovation goals that include user-centered innovation, market innovation, organizational change, and strategic direction (p.749) | - | - |
| R&D Management | Magistretti et al. (2022) | Economic | Examine the role of DT in the early research phase of the technology innovation process and characterize DT practices as a way of bringing a longer term, exploratory perspective in projects (p.118) |  |  |
| Industrial Marketing Management | Magistretti et al. (2021) | Economic | Identify five DT dynamic capabilities to support digital transformation, by helping organizations capitalizing on the market opportunities that digital technologies provide (p.68) | - | - |
| Journal of Product Innovation Management | Magistretti et al. (2021) | Economic | Describe DT as a dynamic capability for innovation, used by organizations to sensing and seizing new business opportunities (p.658) | - | - |
| Long Range Planning | Magistretti et al. (2022) | Economic | Conceptualize DT as a set of practices that needs to be adapted to the nature of the innovation project. Advance and test hypotheses on how selecting the right set of practices can improve the business performance of a project (p.4-5) | - | - |
| Project Management Journal | Mahmoud-  Jouini et al. (2016) | Economic | Propose that DT can make important contributions to managing the explorative phase, managing the involvement of stakeholders in the project, and managing the project in relation to the strategizing process of the firm (p.144) | - Acknowledge that DT has a social dimension related to the ability to imagine the world from multiple perspectives (p.149) | - |
| Research Technology Management | Mahmoud-  Jouini et al. (2019) | Economic | Describe how DT is leveraged by a large, technology-driven firm (p.50) as a way to create new business models and value propositions (p.57) in a competitive innovation environment (p.55) | - | - |
| Small Business Economics | Mansoori and Lackéus (2020) | Economic | Compares DT with effectuation, business planning, and other approaches for new venture development (p.791) | - | - |
| Strategy & Leadership | Martin (2010) | Economic | Presents DT as a source of sustainable competitive advantage, explaining that it allows to balance exploration and exploitation (p.37) | - | - |
| Design Management Review | McCullagh (2010) | Economic | Makes the case that DT is relevant for business leaders (p.36) but it results in competitive advantage when it combines creativity with a high degree of problem solving (p.38) | Declares that sustainability is a hot topic, just like DT (p.36) | Declares that sustainability is a hot topic, just like DT (p.36) |
| Design Management Review | McCullagh (2013) | Economic | Argues that DT can improve business performance by allowing cross-silo communication, boosting experience awareness, foresight and vision, prototyping, problem solving, communication, opportunity identification (p.33-34) | - | - |
| European Management Journal | Meinel et al. (2020) | Economic | Make an experimental study with 53 innovation teams to conclude that Using an experimental design and applying DT results in a superior innovation performance and economic success for the firm (p.669), through product concepts that are more feasible and relevant, but not necessarily more novel (p. 661) | - | - |
| Journal of Product Innovation Management | Micheli et al. (2018) | Economic | Argue that DT consists in elevating design to a strategic function in the organization (p.629), and that this may result in long term financial sustainability and and competitiveness for the organization (p.630) | - | - |
| Journal of Product Innovation Management | Micheli et al. (2019) | Economic, social | Mainly contextualise DT in business as an approach for creative problem-solving and improve innovation performance (p.124-125), to then make a conceptual review to identify 10 attributes (p.132-133) and 8 methods and tools (p.137) of DT, ultimately discussing implication for managers (p.144) | Distills 10 attributes of DT, including aspects that that are relevant for social impact, including wicked problem solving and human centeredness (p.132) |  |
| California Management Review | Mount et al. (2020) | Social | - | Examines how DT can be leveraged as a complex problem-solving approach to benefit society, specifically investigating how young people can achieve an AIDS-free generation (p.104) | Acknowledges the connection between social and ecological issues (p.109) |
| Journal of Product Innovation Management | Nagaraj et al. (2020) | Economic | Discuss DT in the context of new product development, ultimately arguing that DT is a dynamic capability important to expand the organization’s knowledge resource base (p.297) and achieve competitive advantage (p.313) | Acknowledges that DT can result in products that are more useful and novel (p.297) but does not elaborate on social implications | - |
| Journal of Business Research | Nakata and Hwang (2020) | Economic | Introduce DT as an approach that businesses can use to solve human problems and grow sales, and conclude that DT strengthens new product and service performance, and has robust effects across levels of market turbulence (p.117) | Briefly discuss human centeredness and an attribute of DT (p.118) and include in the review a conceptual paper that links design to addressing global sustainability issues, although this study is not specifically about DT (p.119) | Include in the review a conceptual paper that links design to addressing global sustainability issues, although this study is not specifically about DT (p.119) |
| Journal of Business Research | Nakata and Bahadir (2022) | Economic | Conduct a service that shows that design leadership, design inclusion and especially DT have a positive effect on product and service innovaiton. |  |  |
| Journal of Brand Management | Nedergaard and Gyrd-Jones (2013) | Economic | Explain that DT can foster sustainable (in an economic sense) brand-based innovation by: orienting innovation and investments around the brand; thinking on a human scale to generate unique customer insight; considering the current and future scope of firm resources needed to attain and sustain competitive advantages (p.762) | - | - |
| California Management Review | Pitsis et al. (2020) | Economic, social | Present DT as an innovation approach that is very important for all kind of organizations, including businesses, in the transition towards 4th industrial revolution (p.5-8) | Explain that, in the transition towards 4th industrial revolution, DT can be used by businesses but also by the public sector to foster a positive relationship of humans with technology (p.5-8) | - |
| Design Management Review | Porcini (2009) | Economic, social | Discusses how DT can be executed successfully in a multinational company (p.7) as a way to create new technologies and business models (p.8), and grow brand equity (p.9) | States that society and individuals represent a key starting point for the DT process (p.8) | - |
| Journal of Business Strategy | Radnejad et al. (2020) | Economic | Discuss DT as a tool for radical innovation, giving an historical view on how firms in selling watches have leveraged to gain competitive advantage over time. | Recognizes that DT is about human needs, although do not elaborate on this aspect | - |
| Journal of Product Innovation Management | Randhawa et al. (2021) | Economic | Derive a process model of how DT practices can help middle managers to develop ambidextrous innovation portfolios that overcome organizational inertia and maintain competitiveness. | - | - |
| Design Management Journal | Rauth et al. (2014) | Economic | Introduce DT as an essential and profitable (p.55) innovation capability for large organizations (p.47) and explore how it can be implemented in this context (p.52) | - | - |
| R&D Management | Robbins and Fu (2022) | Economic | Conduct a survey showing the adopting DT practices in R&D management boosts organizational innovative performance (p. 712) |  |  |
| Design Management Journal | Rosensweig (2011) | Economic | Discusses DT as a dynamic capability to gain competitive advantage (p.16-25) | In the literature review, briefly cites Schon around the social relevance of DT | - |
| Design Management Journal | Rylander (2009) | Economic | Discusses DT in an economic context, comparing how design firms and knowledge intensive firms solve business problems (p.7) | - | - |
| Journal of Business Strategy | Sato (2009) | Economic | Makes the case that DT allows business firms to deliver great innovations (p.41), increase market share, revenues. Margins and savings (p.45), differentiate and outrun competitors (p.46) | Briefly mentions that DT allows to address the social (p.43) and cultural (p.46) needs of people but does not elaborate upon it |  |
| Design Management Review | Sato et al. (2010) | Economic | Explain how DT can be used to promote organizational change to deliver better business results, discussing the case of HP (p.45) | - | - |
| Journal of Product Innovation Management | Seidel and Fixson (2013) | Social | - | Discuss the application of DT in multidisciplinary teams as a way to create better innovation outcomes that can address user needs (p.19), with cases focusing on improved healthcare (p.24) | - |
| Business Process Management Journal | Snyder et al. (2018) | Social, environmental, economic | Discuss how DT can promote organizational change toward a value-based leadership, which is essential for a business to succeed (p.1298) | Make a strong explicit connection between the use of DT, value-based leadership and positive social values within the organization (p.1289, 1294) | Makes a connection between between the use of DT, value based leadership, positive environmental values within the organization, such as minimizing waste (p.1289) |
| Design Management Review | Sobel and Groeger (2013) | Economic | Discuss opportunities and barriers related to the implementation of DT in Australian businesses (p.26-31), making the case that the government should also play a role in this regard to foster economic development (p.31) | - | - |
| International Journal of Research in Business and Social Science | Soyupak and Humanur (2019) | Social, economic | Explore how DT can support emerging tech-startups to develop innovative ideas (p.59) as a way to foster economic development and job creation in Turkey (p.61) | Explore how DT can support emerging tech-startups to develop innovative ideas (p.59) with a human centered approach to solve various problems including social ones, healthcare related (p-64, 67) |  |
| Journal of Product Innovation Management | Spanjol and Nobles (2020) | Economic | Encourage innovation management scholarly investigation on DT to improve innovation management practice and business performance | - | - |
| Journal of Management Inquiry | Stephens and Boland (2015) | Social | Mentions that DT can be used to find market opportunities and develop a viable business strategy (p.219) | Discussed the nature of DT and how this approach can used to solve complex problems (p.219), using as an illustrative case how a clinic used DT to improve the patients’ experience (p.225) |  |
| California Management Review | Thompson et al. (2020) | Economic, social | Discusses several consultancy projects performed by IDEO to show how using DT can support business in different ways such as understanding the way consumers shop to better compete for their attention (p.87), how to make customers aware of product quality in the meat industry (p.91), how to improve in-flight services (p.95) | Discusses the case of an IDEO project aimed at improving the quality of life of people suffering from chronic diseases (p.89) | - |
| Journal of Business Research | Vendraminelli et al., 2022 | Economic | Frame DT as an approach for planning and executing digital transformation strategies (p.1) and use a case study to show how DT tools and practices support in identifying digital opportunities that are aligned with business success indicators (p.6). |  |  |
| Marketing theory | Venkatesh et al. (2012) | Economic,  social | Discuss DT in the context of marketing strategy arguing that it is essential to maintain competitive advantage (p.290) | States that using DT results in the creation of customer value, ultimately as a way to enable people to engage in their individual or social endeavors, whether these endeavors be utilitarian, functional, material, communicative, symbolic, or experiential (p.291) | Interview company managers on their design philosophy and, in one case, make a mention to a deep concern for environmental issues (p.294) |
| Journal of Product Innovation Management | Verganti et al. (2020) | Economic, social | Examines how DT and the design process within business organizations, including Netflix, AirBnB, Microsoft and Tesla, changes when it is not performed by humans but by artificial intelligence instead (p.212), ultimately arguing that this will bring about a transformation in the economy and drawing implications for managers wanting to create value (p.225) | Reflects on the implication of delegating problem solving to machines instead of humans, and about the potential consequences for human labor (p.212-226) | - |
| Journal of Product Innovation Management | Verganti et al. (2022) | Economic | Discuss how DT has grown as a problem-solving approach increasingly used by organizations in order to ensure value creation (p.605). | - | Call for system thinking as a way of extending the potential of DT for addressing long-term and complex social and environmental challenges (p.618) |
| MIS Quarterly Executive | Vetterli et al. (2016) | Economic | Discuss how DT can be used to foster customer centricity in the banking sector (p.37), which is in turn essential for creating a competitive advantage for banks through innovative offerings (p.52) | - | - |
| Design Management Review | Vogel (2009) | Economic, social, environmental | Provides a historical evolution of DT highlighting its relevance for business, innovation and economic development, mentioning the competitive aspects related to using design strategically (p.17-27) | Highlights the relevance of DT for building a kinder, more humane business that connects to social value systems in diverse parts of the world, including emerging countries (p.26) | Highlights the relevance of DT for building a kinder, more humane business that integrates environmental responsibility into its products (p.26) |
| International Journal of Management and Innovation | Wattanasupachoke (2012) | Economic | Examines DT as an approach for business to generate competitive strategies and improve performance | - | - |
| Journal of Management education | Welsh and Dehler (2013) | Social | - | Investigate how DT can be used in management education to foster critical reflection, multiliteracy, the ability to negotiate identity, engage in constructive critique, become skilled in knowledge transformation, and create tangible and implementable outcomes (p.771), as a way to improve society (p.773) | - |
| California Management Review | Wrigley et al. (2020) | Economic, social | Introduce DT as the foundation of competitive advantage for an organization (p.127), and investigate the organizational conditions needed to implement this approach (p.125) within 8 companies, who are mostly aiming to grow and maximise profit (p.130) | Discusses cases where companies are using DT to make a positive social impact, for example implìroving elderly care (p.130, distributing electricity in remote areas (p.130) and preventing child injuries (p.131) | - |
| Harvard Business Review | Yoo and Kim (2015) | Economic | Explains how Samsung was able to use DT as a way to compete in the consumer electronic industry and increase profit margins | - | - |
| Management Decision | Zheng (2018) | Economic | Discusses DT as an approach for business firms to gain competitive advantage (p.747) by improving strategy through ambidextrous learning (p.736) | Briefly mentions that using DT for ambidextrous learning in the context of business organizations also entails considering society and the impact on communities (p.741) | Briefly mentions that using DT for ambidextrous learning in the context of business organizations also entails considering the environment (p.741) |

**APPENDIX B**

**Theoretical linking between reflexivity and framing, and relevance for responsible policies**

| **Author(s)** | **Theoretical linking between reflexivity and framing** | **Relevance for responsible policies** |
| --- | --- | --- |
| Aukes et al. (2018) | Discuss framing as *“interactional and interpretive mechanism”* for reflection, where an actor attempts *“understanding the ideas, motives, and concerns of others”* on a certain issue for creating his *“own definition of problems and solutions”* (title; keywords; abstract; p.409) | Outline the figure of the “policy entrepreneur”, who takes the responsibility to foster the transition toward sustainable development. This is illustrated with the case of the policy process underlying the designation of a land area to *“coastal protection, nature development as well as recreational purposes”* (title; abstract) |
| Beckman (2020) | Presents framing as an activity that *“sits at the intersection between reflective observation and abstract conceptualization”* used for *“more in-depth exploration of a situation and unpacking of the messy data”* (figure 1; p.47) | Does not elaborate specifically on responsibility and policy making issues, but makes the case that framing should not be studied too narrowly, focusing only on how businesses apply it, looking at it also in other contexts, such as *“governmental departments”* (p.58) |
| Hey et al. (2007) | Make the case that framing is an activity that occurs in the *“early phases”* of a design process, where multiple actors reflect together on their individual ideas to *“negotiate”* a common point of view (figure 1; p.82) | - |
| Paton & Dorst (2011) | Highlight that *“the ability to frame a problematic situation in new and interesting ways is widely seen as one of the key characteristics of design thinking”* and that *“reframing can also occur as a result of reflection, throughout the design process”* (p. 573, 575) | Make the case that “*framing is not a concept that is unique to design theory”*, being also present in *“policy studies”,* but make no explicit mention to the responsibility aspects within (p.574) |
| Vliegenthart & Roggeband (2007) | Present framing as a *“divergent”* activity to reflect and “group ideas”. This is *“important in shaping the debate”*because it *“provides space”* for *“multiple and contrasting perspectives on a certain issue”* (title; p.526, 530, 538, 541) | Use the case of *“the public debate on migration in the Dutch parliament”* to illustrate the relevance of *“framing in parliament”,* arguing that *“state policies and arrangements should help migrants in their integration and emancipation process”* (title; abstract; p.531) |
| Schon (1983) | Explains that since innovators always deal with new and unique situations, which cannot be dealt with using standard theories and techniques, they must *“reflect in action”* to *“construct an understanding”* of the problematic situation and *“reframe it”* in order to address it (title; p.129) | - |
| Schon & Rein (1994) | Explain that framing allows to reflect *“by focusing attention of different facts and by interpreting the same facts in different ways”,* to ultimately deal with *“complex situations”* that have no *“right or wrong solution”* (title; p. 28, 33) | Argue that *“reflective framing”* is most useful when applied to *“make sense of intractable policy controversies”* and create new *“policy frames”* that make a positive impact on society, for example targeting the*“sources of poverty and crime”* (title; p. Xvii, 5, 22, 33) |
| Seidel & Fixon (2013) | Explain that *“team reflexivity refers to the degree to which individuals collectively reflect upon their actions and processes”*, debating ideas supported by previously collected information, which can be understood as framing (p.29) | - |
| Stilgoe et al. (2013) | Explain that *“reflexivity means holding a mirror up to one’s own activities, commitments and assumptions, being aware of the limits of knowledge and being mindful that a particular framing of an issue may not be universally held”* (p.1571) | Argue that this type of reflective framing is a core *“dimension”* of responsible innovation and a *“public matter”* taking place at the level of *“institutional practice”* (abstract; table 2; p.1571) |
| Windahl et al. (2020) | Present framing as an *“intentional and system-oriented”*activity used to reflect and *“think outside”* the box in order to create a new problem space, as mapped on the double diamond model (figure 1; p.5) | Explain that this type of framing can also be used to*“raise purpose questions”* and foster *“institutional change”* toward *“sustainability and shared prosperity in the form of individual and communal well-being”* (p.9) |

**Theoretical linking between anticipation and envisioning, and relevance for responsible project ideas**

| **Author(s)** | **Theoretical linking between anticipation and envisioning** | **Relevance for responsible project ideas** |
| --- | --- | --- |
| Cagnin (2018) | Emphasizes the importance of an *“applied anticipatory systems approach to using the future”* as it *“provides policy and decision makers as well as individuals with an enhanced capacity to both question and invent the anticipatory assumptions that inform their choices*” (p. 525) | Details how foresight and futures literacy enabled by “*anticipation*” and *“imaginary futures*” are critical for new and disruptive project ideas. Thereby, the approach considers the wider institutional context and its implications for a better collective future, requiring responsible considerations such as in the example of “*more sustainable, human and secure cities, with the generation and use of clean and renewable energy*” (p. 529) |
| Daalsgard (2014) | States that designers seek to “*envision and explore potential futures and to expose potential future users to their concepts to evaluate which course to take in the design process*” (p. 146). Anticipation of user needs and exposure to future scenarios is thus central to the process | - |
| Fuller (1957) | Proposes the idea of a *“comprehensive anticipatory design science”* to craft a *“global vision”* for the future of humanity | Explicitly links the *“comprehensive anticipatory design science” and “global vision”* to sustainable development problems that can be solved by new project ideas for *“planning innovation”*. |
| Joly et al. (2019) | Bring forth the argument that “*at a macro level of service ecosystems, a design perspective contributes to enable institutional change, by envisioning new services ecosystems that support more sustainable lifestyles and consumption”* (p. 681), anticipating different or better futures | Enabled by such envisioning approaches and drawing on past research, the authors argue that design is crucial for “*enabling society-driven innovation, by addressing social challenges and creating solutions that support more sustainable service ecosystems*”, including “*a process for radical change through the envisioning and design of new service systems*” (p. 705) |
| Karpen et al. (2017) | Discuss “*envisioning*” by way of “*creating future scenarios and using these as departure points for developing design solutions and future service experience settings” (p. 391),* and in so doing highlight the need for anticipating or “*imagining of what might be rather than what is” (p. 395)* | Inform an understanding of design-driven innovation that aims at systemic “*betterment*” (p. 391), “*envisaging future scenarios and anticipating new needs, respective solutions*”, while “*facilitating more sustainable relationships*” with a “*commitment to responsible services and interactions*” (p. 392) |
| Kyffin & Gardien  (2009) | Underline the need for “*futures and foresight, which involves identifying socio-cultural trends and narratives to identify emerging value and needs”*, applying “*scenarios and narratives, which serve as input for the experience prototypes (*probes)” to explore new territories and anticipate meaningful solutions (p. 62-63) | Argue “*that transferring innovation directly into new business activities is not necessarily the best way forward*” in view of responsible innovation, but instead to probe and test “*imaginative ideas*” or scenarios (p. 59) first to avoid resource wastage and build a foundation for “*innovation towards a better future quality of life*” (p. 62) |
| Micheli et al. (2018) | Refer as part of their literature study to the benefits of *envisioning potential futures* (cf. p. 134; 137)*,* guiding strategic directions by anticipating potential barriers and success factors | - |
| Miettinen (2009) | “*Focusing too much on current reality makes it problematic to envision a world that is different.Communicating the future with scenario tools helps the various stakeholders to discuss the service opportunities, but for implementing the new service process, new tools, such as training, consulting, or new service channels, may be needed”* (p. 76) | Illustrates the role of scenario-based design, among other techniques, describing “*current or envisioned work practises or tasks of the users and thus help different stakeholders (including the users themselves) understand the tasks in their contexts, evaluate the practises and suggest changes to these practises*” (p. 22). The author thus recognizes the need to anticipate or “*predict how people could act in particular situations*” or contexts (p. 22), to be able to make assessments of solutions across various criteria such as in terms of desirability or sustainability |
| Morelli  (2007) | Demonstrate the relevance of design orienting scenarios (DOS), which aim *“to generate visions of the future that are subsequently orienting operative design decisions”,* particularly in the context of “social innovation” and anticipating the design consequences of “*single decisions*” for the focal system or even specific actors, “*through one or more visions of this particular focused system*” (p. 14) | Contend that “*DOS are aimed at generating a plurality of hypotheses involving local actors, possible users, and other stakeholders in the development of the scenarios*” (p. 14). This mechanism for anticipating and envisioning future states is presented to be critical particularly for notions of responsible innovation as measured in terms of “*social quality*” outcomes, or citizens ability to actively participate in and contribute to value creation and community life (p. 6) |
| Windahl et al. (2020) | Present the need for innovators to be “*anticipating and encouraging more systemic impact*” across different levels of the ecosystem (p. 7), which requires them to envision different future scenarios as part of this process | Explain that systemic impact is facilitated by envisioning and anticipating the consequences of new solutions across stakeholders and levels of analysis (micro, meso, macro, meta) for “*individual and communal well-being*” (p. 9) |

**Theoretical linking between inclusion and co-creating, and relevance for responsible coalitions of organizations**

| **Author(s)** | **Theoretical linking between inclusion and co-creating** | **Relevance for responsible coalitions of organizations** |
| --- | --- | --- |
| Barnes et al.  (2017) | Showcase the role of inclusive urban design in the context of changing human practice of transportation behavior toward greater adoption of cycling. The authors summarize that “*through co-creation a set of stakeholders can share individual practices, discuss social boundary conditions and their legitimacy, collectively innovate and break existing social practices into its elements*” (p. 3311), make the need for inclusive designerly approaches central | Validate the important role of inclusion and co-creation specifically for responsible innovation, in this case a transition of a social system toward more sustainable transportation. To change such social systems, the authors argue for the need to involve various stakeholders across organizations to challenge and reimagine social practices enacted by those focal stakeholders. |
| Gemser & Perks (2015) | Highlight the role of “*customer co-creation. Here, customers, and possibly other actors, are actively involved in the innovation process of a firm*” (p. 660)’ going beyond a “*community of similar customers, toward a complex system where a multitude of both businesses and end customers interact and innovate”* (p. 662). | - |
| Manzini (2017) | Argues that in the contemporary fluid world the “(co)creation of new social forms” is an effort that must *“include various kind of community that make up the connective tissue of every society and thus play the fundamental role of making it cohesive, ductile and, ultimately, resilient” (abstract; p.188)* | Makes the case that “designing coalitions” of organizations are responsible for *“(co)creating”* a better society that is more *“cohesive, ductile and, ultimately, resilient” (abstract; p.188)* |
| Melles et al. (2011) | Suggest that “*participatory design* [...] *should be fundamental to socially responsible design,* [and]*displaces the expertise and authority of the designer*” (p. 147-148). In doing so, the authors argue for the need to include and leverage stakeholder knowledge, with the designer being a “facilitator” enabling “*co-creation [as] a new addition to the socially responsible design toolkit*” (p. 148) | Display inclusion and co-creation as key ingredients for responsible design. For example, the authors state that “*the most successful examples of socially responsible design have emerged from co-design where designers have engaged effectively with communities and then co-designed and co-manufactured a solution that utilises local or regional materials, craftsmanship and expertise, facilitates new skills and knowledge acquisition, empowers the community and allows the user to ‘own’ the solution*” (p. 149) |
| Morelli  (2007) | Make a plea for *“local systems of innovation*” whereby “*networks of actors directly or indirectly participating in the development of solutions*. *The identification of the actors is critical to explore the system of interests, skills, and (tacit and explicit) knowledge that can be mobilized”.* | Make an explicit link between inclusive and co-creative design and socially responsible innovation. For example, the authors state that to achieve social quality as desired responsible outcome, this “*implies the inclusion of those parts of the society (especially in developed countries) that otherwise are excluded by social life, and those communities (mainly in developing countries) whose consistency is undermined by poor socioeconomic conditions, which limit the individual’s range of possible actions to a mere fight for subsistence*” (p. 6). This in turn requires the involvement of those communities in the innovation process to make solutions locally and contextually meaningful, with a changed role of the designer to becoming “*facilitators of a system of value co-production*” |
| Patricio et al. (2020) | Building on past literature, the authors refer to the role of inclusive and co-creative design in the context of healthcare systems, such that it “*contributed toward developing more inclusive, efficient and integrated care”*and *“proved effective in engaging and co-designing with people who have different forms of disabilities and vulnerabilities*” (p. 6) | - |
| Smith & Iversen  (2018) | Present an approach of participatory design that is both collaborative and inclusive, with a “*move away from engagement of limited stakeholders in preconfigured design processes and predefined technology outcomes, towards more complex and long-term engagement with heterogeneous communities and larger ecologies of social and technological transformation*” (p. 9) | Offer participatory design that is particularly relevant to responsible innovation given the diversity and extended involvement of stakeholders, to realize benefits that make a difference to people both on scale and in the long-term |
| Thorpe & Gamman (2011) | Call for “*socially responsible design*” that builds on a philosophy of ‘designing with’ rather than ‘designing for’. For this purpose, the authors argue for “*collaboration*” and leveraging stakeholders’ *“agency*” in this process, and that “*equitable arrangements between stakeholders are essential to ensure the successful delivery of design for social change in the real world*” | Outline that “*robust socially responsive design and innovation methodologies and engagement strategies are essential, and need to be mindfully applied by designers seeking to make effective and appropriate contributions to meeting social goals in new, sustainable ways*” (p. 217), prescribing “*co-design processes inclusive of diverse actors and agenda*” (p. 220) |
| Voegtlin & Scherer (2017) | Discuss *“inclusion”* in terms of global solutions *“co-created”* by multiple stakeholders (p .231, 237) | Argue that between inclusive co-creation is about *“global governance schemes”* supporting responsible innovation for sustainable development performed by coalitions encompassing *“governments”*, *“the civil society and representatives of local communities”*, *“business actors”*as well as *“NGOs and environmental agencies”* (p. 237, 239, 240) |
| Voorberg et al. (2014) | Define “*social innovation as the creation of long-lasting outcomes that aim to address social needs by fundamentally changing the relationships, positions and rules between the involved stakeholders, through an open process of participation, exchange and collaboration with relevant stakeholders*” (p. 1334), which represents an inclusive and co-creative design process | Focusing on “*social innovation*”, the authors argue that “*policy makers and politicians consider co-creation/co-production with citizens as a necessary condition to create innovative public services that actually meet the needs of citizens, given a number of social challenges, like ageing and urban regeneration, and all of this within the context of austerity. Hence, co-creation/co-production seems to be considered as a cornerstone for social innovation in the public sector*” (p. 1346) |

**Theoretical linking between responsiveness and prototyping, and relevance for responsible products and services**

| **Author(s)** | **Theoretical linking between responsiveness and prototyping** | **Relevance for responsible products and services** |
| --- | --- | --- |
| Ampatzidou & Gugerell (2018) | Unpack the role of prototyping and being responsive in a participatory game design innovation process. Importantly, the authors introduce “*participatory prototyping*”, “*particularly for investigating real-world complex issues that demand the involvement of various stakeholders*” (p. 348). In so doing, they “*give shape*” (p. 348) to future solutions and/or their components that are tested and modified along the way based on the learnings achieved | Exhibit how “*participatory prototyping*” can be an effective means for responsible innovation, as showcased in the context of developing serious games for future energy systems and “*translating complex real-world issues*” into a format that is responsive to solving problems such as transitioning to renewable energy |
| Baldassarre et al. (2020) | Illustrate the role of being responsive and responsible through experimentation. The authors argue that “*piloting a prototype forces organizations to simultaneously consider the desirability (i.e., what users want), feasibility (i.e., what is technically achievable), viability (i.e., what is financially possible), and sustainability (i.e., what is economically, socially and environmentally acceptable) of a new business model*” (p. 1), while building a more agile approach into the development process | In the context of *sustainable business model innovation*, the authors show how more responsible solutions can be found through “*small-scale pilots as a first implementation step*” (p. 1), yet these small-scale pilots conserve resources (compared to full scaling or implementation), while offering the possibility to further adjust the shape or direction of innovation |
| Ceschin & Gaziulusoy  (2016) | Building on extant literature, the authors summarize that “*systemic thinking, alongside more conventional design skills such as visualization and prototyping are considered as strengths of a design approach in achieving social innovation*” (p. 134) | As the authors do not focus on prototyping but on design for sustainability overall, the role of prototyping is not further advanced beyond the acknowledgement that prototyping has a role to play to foster responsible outcomes through design, yet call for further research that seeks to better understand the role of experimentation in product service systems for such outcomes |
| Geissdoerfer et al. (2016) | Condense that “*prototyping is concerned with investigating and enriching different solution ideas by repeatedly building and discarding low-resolution and rapid prototypes of early conceptualizations*” (p. 1220). The ‘repeated’ nature thereby encapsulates a learning process that enables design-driven innovation to respond to early feedback | Argue that “*these characteristics of design thinking* [and prototyping in particular] *could be utilized to enhance the sustainable business modelling proces*s” (p. 1220). For example, to identify “*major problems for implementation*” that are associated with the innovation of a focal sustainable business model (p. 1228) |
| Hillgren et al. (2011) | Claim that “*different types of prototype are used in social innovation*”, including both “*fast prototyping*” and “slow prototyping”, depending on contextual circumstances and purpose. This enables organizations to either move relatively “*quickly*” and cheaply, or to organically develop and “*extensively test*” solutions as a process. Either prototype enables innovators to remain “*adaptable to accommodate*” local and emerging needs (p. 173). | Highlight the importance of responsible innovation in the form of “*social innovation, which is aimed at developing new ideas and solutions in response to social needs*”. Building on extant research, the authors mention that in these complex environments “*prototyping should be performed through co-creation*” and in context (p. 173-174), ensuring that “*future roles and resource flows*” are fit for purpose and resourceful, while *revealing* “*opportunities and dilemmas*” (p. 179) |
| Kleinsmann & ten Bhömer (2020) | Introduce new types of prototyping (service interface proto-trial; service prototype) that enable designers and the design process to be more responsive by “*exploring several options*” and stimulating “*collaborative creation [...] in an early stage*” (p. 65). | Develop the new types of prototyping in the context of “*smart textile service for people who suffer from dementia*” (p. 68) in collaboration with a diverse set of stakeholders, and empirically demonstrate the role that prototyping, including traditional and the newly developed types, play for responsible innovation. |
| Murray, Caulier-Grice, & Mulgan (2010) | Reveal the role of experimenting and prototyping to be responsive, as “*it’s through iteration, and trial and error, that coalitions gather strength (for example, linking users to professionals) and conflicts are resolved (including battles with entrenched interests)’*” and for the “*measures of success come to be agreed upon*” (p. 12) | Create a direct link between the need to prototype and being responsive in that way to social innovation, as a form of responsible innovation. As a result, “*the process of refining and testing ideas is particularly important in the social economy*” and with that to tackle some of the “*biggest challenges of our times*” (p. 2) |
| Reay et al. (2016) | “*Propose that prototyping refers not only to evolving design outputs, but also to the space in which they are made, which may itself be only the most recent iteration of an ongoing co-design process that includes multiple stakeholders*” (p. 229). With this foundation, the authors experiment with the development of a co-design space, the Design for Health and Wellbeing Lab, for the purpose of better responding to emerging healthcare and design needs | Highlight that “*as sites dedicated to addressing ‘Wicked Problems’ through collective problem solving, social labs are becoming central to this shift, and their emphasis on rapid prototyping not only helps demonstrate the viability of their solutions, but also helps facilitate collaboration*” (p. 229). In combination, these characteristics render responsive prototyping particularly valuable in view of responsible innovation and solving wicked problems |
| Sato et al. (2010)    (DMIR) | Promote the need for prototyping” *to make organization change and development more responsive*” (p. 45), as “*prototyping ideas make concepts concrete, so the concepts that embody the design principles can be shared and evaluated in real-world settings. What is learned from failures often hints at what will work* [or not]” (p. 47) | While the authors do not explicitly refer to responsible innovation and change per se, they highlight that “*iteratively developing, prototyping, and validating critical portions of a system can be more cost- and time-effective, and less risky, than doing an all-or-nothing, full-blown launch*” (p. 47). Prototyping is thus viewed as a means for more resourceful innovation, which also benefits the innovation process through greater responsiveness |
| Viswanathan & Sridharan (2011) | Seek to understand success factors for “*concept and prototype development”* in “base of the pyramid” (BoP) markets. Among these factors, the authors find “*design for customization*” (p. 62-63) a core element, which highlights the need for being responsive to local or even individual needs (e.g. responding to different local languages for concept and prototype development; modular rather than integral solution structures; different formats or modes of delivery etc.), for example to “*accommodate the “fine-tuning” of product delivery by local stakeholders* | Present a view that prototypes are central to radical innovation that here classifies as being responsible in that it aims to change the lives of many people living at the base of the socio-economic pyramid for the better. This might be achieved through responsible solutions that are “*either critical to survival in the current term or critical to transformational progress in the long term or both*”, while ensuring “*local sustainability*” (p.66) |
